# Abrupt declines in marine phytoplankton production driven by warming and biodiversity loss in a microcosm experiment

**DOI:** 10.1111/ele.13444

**Published:** 2020-01-11

**Authors:** Elvire Bestion, Samuel Barton, Francisca C. García, Ruth Warfield, Gabriel Yvon‐Durocher

**Affiliations:** ^1^ Environment and Sustainability Institute University of Exeter Penryn TR10 9EZ UK; ^2^ Station d'Ecologie Théorique et Expérimentale UMR 5321 Université Paul Sabatier Moulis 09200 France

**Keywords:** Climate change, biodiversity loss, phytoplankton, biodiversity–ecosystem functioning, thermal performance curve

## Abstract

Rising sea surface temperatures are expected to lead to the loss of phytoplankton biodiversity. However, we currently understand very little about the interactions between warming, loss of phytoplankton diversity and its impact on the oceans' primary production. We experimentally manipulated the species richness of marine phytoplankton communities under a range of warming scenarios, and found that ecosystem production declined more abruptly with species loss in communities exposed to higher temperatures. Species contributing positively to ecosystem production in the warmed treatments were those that had the highest optimal temperatures for photosynthesis, implying that the synergistic impacts of warming and biodiversity loss on ecosystem functioning were mediated by thermal trait variability. As species were lost from the communities, the probability of taxa remaining that could tolerate warming diminished, resulting in abrupt declines in ecosystem production. Our results highlight the potential for synergistic effects of warming and biodiversity loss on marine primary production.

## Graphical abstract

Climate change and biodiversity loss can interact to drive marine primary production. We experimentally manipulated the species richness of marine phytoplankton communities under a range of warming scenarios, and found that ecosystem production declined more abruptly with species loss in communities exposed to higher temperatures.

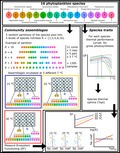

## Introduction

Experiments, mostly in grasslands, have shown strong effects of plant diversity on ecosystem production (Naeem *et al*. 1994; Tilman & Downing 1994; Tilman *et al*. 1997; Loreau *et al*. 2001; Hooper *et al*. 2005). Production typically increases with species richness in a saturating manner or as a continuously increasing, but decelerating function (i.e. logarithmic), implying some degree of functional redundancy among species (Reich *et al*. 2012). The shape of the diversity–production relationship has important implications for understanding the impacts of biodiversity loss on ecosystem function. If the diversity–production relationship is steep and saturates slowly, then the loss of even a few species from diverse communities could have marked impacts on ecosystem function (Reich *et al*. 2012).

In spite of the fact that they contribute nearly half of global primary production (Falkowski 1994; Field *et al*. 1998), the relationship between biodiversity and ecosystem functioning in marine phytoplankton is poorly understood (but see Ptacnik *et al*. 2010 for a summary of current understanding). What we do know about the links between biodiversity and ecosystem functioning in marine phytoplankton mostly come from observational studies. For example, global patterns of marine phytoplankton biodiversity tend to show a unimodal relationship between species richness and ecosystem production (Irigoien *et al*. 2004), although even this result has been challenged due to methodological concerns (Cermeño *et al*. 2013). Models suggest that the unimodality could be due to stronger top‐down control by grazers at extreme levels of ecosystem production – the so‐called ‘kill the winner hypothesis' (Vallina *et al*. 2014). Analyses have demonstrated positive associations between ecosystem production and community‐level diversity in cell size (Acevedo‐Trejos *et al*. 2018), as well as complex interactions between production and traits linked to grazing and nutrient uptake (Prowe *et al*. 2012a,b; Cermeño *et al*. 2016; Hodapp *et al*. 2016). In freshwater ecosystems, primary production and resource use efficiency have been found to be log‐linearly related to taxonomic richness (Ptacnik *et al*. 2008, Striebel *et al*. 2009). Nevertheless, the mechanisms underpinning patterns of phytoplankton biodiversity and ecosystem production are poorly understood in both marine and freshwater ecosystems largely due to a dearth of controlled experiments.

Even less is known about the links between biodiversity and production in the face of environmental change. Recent work has shown that environmental change (e.g. warming, elevated CO_2_, nutrient pollution, drought) can alter both diversity, ecosystem production and the relationship between diversity and production, though the mechanisms underlying these changes are often unclear (Reich *et al*. 2001; Lewandowska *et al*. 2012, 2014; Steudel *et al*. 2012; Isbell *et al*. 2015; Striebel *et al*. 2016). The insurance hypothesis and the ‘portfolio effect’ propose that biodiversity will be important for maintaining ecosystem functioning in the face of rapid environmental change (Doak *et al*. 1998; Tilman 1999; Yachi & Loreau 1999). Because species inherently differ in their ability to tolerate abiotic change (McGill *et al*. 2006), higher biodiversity provides greater insurance that some species will have traits that enable them to maintain high levels of production and contribute to ecosystem functioning in adverse conditions (Hooper *et al*. 2005). Thus, when environmental change exceeds the tolerance limits of some species but not others, the diversity–production relationship is expected to become steeper and saturate more slowly because communities with fewer species will have reduced probability of including those with traits that enable them to cope with the novel environment, and ecosystem production could decline rapidly with biodiversity loss. Indeed, recent work with heterotrophic bacteria has shown that as temperatures depart from ambient conditions (either via warming or cooling) functional redundancy rapidly declines leading to steeper, less saturating diversity–production relationships (García *et al*. 2018). In phytoplankton, a recent model showed that functional diversity in both thermal and nutrient traits positively affected ecosystem production, with a stronger impact of diversity in thermal traits (thermal optima) than in nutrient traits (Vallina *et al*. 2017). However, to our knowledge, there exist no studies that have experimentally manipulated biodiversity of marine phytoplankton in a climate change context.

Thermal tolerance curves for phytoplankton exhibit characteristic unimodality and left‐skew, where fitness declines more sharply above the optimum than below (Schaum *et al*. 2017; Padfield *et al*. 2017). Given the large interspecific variability in thermal tolerance among phytoplankton (Thomas *et al*. 2012, 2016; Boyd *et al*. 2013; Barton & Yvon‐Durocher 2019) and the importance of thermal tolerance for species interactions (Bestion *et al*. 2018a), we hypothesised that when warming drives temperatures above the thermal optimum for some species but not others, the slope of the relationship between biodiversity and ecosystem functioning should become steeper because more diverse communities will have a higher probability of including species that are able to tolerate warming and maintain ecosystem function as temperature rises.

**Figure 1:**
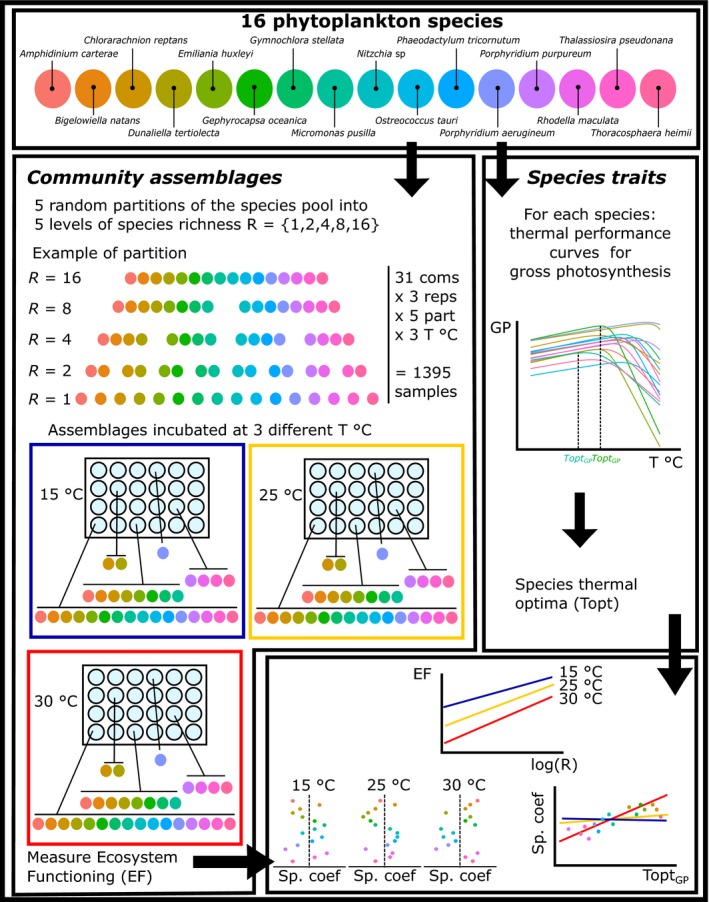
Flow chart of the experimental design.

We tested this hypothesis by experimentally manipulating the species richness of marine phytoplankton communities at a low (15 ºC), intermediate (25 ºC), and high (30 ºC) temperature, and quantifying the impact on ecosystem production in laboratory microcosms. We used 16 species of marine phytoplankton encompassing most of the biogeochemically and ecologically important groups (Diatoms, Dinoflagellates, Coccolithophores, Rhodophytes, Chlorophytes and Prasinophytes, Table S1) and applied a random partitioning experimental design (Bell *et al*. 2009) to create communities with different levels of species richness (Fig. 1). This experimental design allowed quantifying the impacts of species loss on ecosystem functioning as well as evaluating the relative contribution of each species to ecosystem production. To test whether changes in the diversity–functioning relationship could be attributed to species‐level thermal trait variance (as expected under the insurance hypothesis (Doak *et al*. 1998; Tilman 1999; Yachi & Loreau 1999)) we measured the thermal performance curves for photosynthesis for each species and assessed whether species' relative contribution to ecosystem functioning was linked to their photosynthetic thermal tolerance (Fig. 1).

## Method Summary

### Species and culture conditions

The experiment was conducted with 16 marine phytoplankton species sourced from culture collections, *Amphidinium carterae*, *Bigelowiella natans*, *Chlorarachnion reptans*, *Dunaliella tertiolecta*, *Emiliania huxleyi*, *Gephyrocapsa oceanica*, *Gymnochlora stellata*, *Micromonas pusilla*, *Nitzschia* sp., *Ostreococcus tauri*, *Porphyridium aerugineum*, *Porphyridium purpureum*, *Phaeodactylum tricornutum*, *Rhodella maculata*, *Thoracosphaera heimii* and *Thalassiosira pseudonana*. These strains varied widely in their geographic provenance, from the North Atlantic (most strains) to the Mediterranean Sea and the West and South Pacific (Table S1). Species were maintained in semi‐continuous culture in an Infors‐HT shaking incubator (65 rpm) at 20 °C on a 12 : 12 light–dark cycle with a light intensity of 45–50 µmol m^−2^ s^−1^ in K + Si medium (see Supplementary Methods).

### Thermal performance assays

We characterised acute thermal performance curves for gross photosynthesis for each of the 16 species (Fig. 1, see Supplementary Methods for more details). Acute thermal performance curves characterise immediate responses to temperature change and quantify the impacts of temperature on the performance of the photosynthetic machinery. Here we use these measurements as a proxy for the relative difference in thermal tolerance between the 16 taxa. We measured photosynthesis and respiration from 7 to 49 °C with a Clark‐type oxygen electrode as part of a Chlorolab 2 system (Hansatech Ltd, King's Lynn, UK). Samples were taken during the mid‐log growth phase, concentrated to yield clear biomass to detect a sufficient signal of O_2_ flux, and acclimated for 15 min to the assay temperature before measuring photosynthesis and respiration.

Rates of net photosynthesis, measured as O_2_ evolution, were collected across a range of light intensities from 0 to 1800 µmol m^2^ s^−1^. We then used a photosynthesis–irradiance curve at each assay temperature to estimate light‐saturated net photosynthesis *NP_max_*. Respiration (R) was measured in the dark, as oxygen consumption, over a 3‐min period directly following the light response. We calculated gross photosynthesis as *GP* = *NP_max_* + *R* and converted rates to µg O_2_ cell^−1^ h^−1^ after quantifying cell density through flow cytometry (BD Accuri C6).

We quantified the thermal performance curve of gross photosynthesis rates using the modified Sharpe‐Schoolfield equation (Sharpe & DeMichele 1977; Schoolfield *et al*. 1981), which enabled us to estimate the thermal optimum from the resulatant parameters (see Supplementary Methods).

### Biodiversity‐function experiment

Artificial communities for the biodiversity‐functioning experiment were designed using the random partition design described by Bell *et al*. (2009). We randomly divided species into communities with increasing species richness levels from 1, 2, 4, 8 and 16 species, where for each species richness level, the community assemblages were constructed by sampling the 16 species without replacement (Fig. 1). This allowed each species to be represented an equal number of times at each richness level. This process was repeated to form 5 independent partitions of the species pool, so that for each richness level (R) the number of assemblages was 5 × 16/R. Each assemblage was then replicated three times. Further, all replicated communities were subjected to three temperature treatments, 15, 25 and 30 °C, giving for the experiment as a whole a total of 3 × 3 × 5 × (16 + 8 + 4 + 2 + 1) = 1395 communities.

The biodiversity‐function experiment was done in sixty 24 well plates filled with 2 mL of K + Si medium. Each well was inoculated with 1600 cells mL^−1^ of each community (i.e. from 100 cells mL^−1^ per species in the case of 16‐species communities to 1600 cells mL^−1^ per species for monocultures). Samples were grown in three Infors‐HT shaking incubators at 15, 25 and 30 °C on a 12 : 12 light/dark cycle. Distilled water was added every 5 days to refill evaporative water loss. After 19 days, 100 µL samples from each community were taken on a 96 well plate, preserved with 10 µL of 1% sorbitol and frozen at −80 °C after one hour of dark incubation. Cell density was determined by flow cytometry (BD Accuri C6) counting 20 µL on slow flux settings.

### Data analyses

We extracted cells counts and cytometric properties from FSC files with the Bioconductor FlowCore package in R v3.4.2 (R Core Team 2014). Data were filtered to remove values where either log_10_(FSC) < 5, log_10_(SSC) < 5 and/or log_10_(FL3) < 3.5, which are below minimum values observed for live cells of these species. We derived cell chlorophyll *a* content (pg cell^−1^) from FL3 values using the calibration curve described in Supplementary Methods. We calculated community abundance (cells mL^−1^) and total chlorophyll *a* content (sum across all cells per mL). These two metrics were used as proxies for ecosystem production, as found in other studies (Boyd *et al*. 2013). We focus on chlorophyll *a*, as it is the most widely used proxy for studying phytoplankton biomass (Field *et al*. 1998; Marañón *et al*. 2014), but show that the results are largely consistent when using total community abundance (Tables S3–S4, S6, S8, S10 and S12; Figs S1, S3, S4, S5b, S6b and S7b).

The biodiversity–ecosystem functioning (B–EF) relationship was analysed using the analysis of variance method described by Bell *et al*. (2009). This entailed sequentially adding terms to the model and testing whether the additional model complexity improved the fit of the model to the data using AIC. In the model, log‐ecosystem production was treated as the response variable, temperature was included as a factor with three levels (15, 25 and 30 °C) and log‐species richness was included as a covariate. The most parsimonious model included temperature, species richness and their interaction (Table 1, Table S3). We tested for differences in the slope of the BE–F relationship between temperature levels with post hoc contrasts using the ‘lsmeans’ package with tukey *P*‐value adjustment (Tables S2 and S4). We then extracted the residuals from relationships between ecosystem functioning and species richness for each temperature treatment and fitted these residuals to the presence–absence status of each of the 16 species. The species coefficients provided by this method indicate the effect of each species on ecosystem production relative to an average species, where positive values indicate above average contributions and negative values below average contributions (Figs S2 and S3). We then used linear regressions at each temperature level to test whether the species coefficients were correlated to species' photosynthetic thermal optima (Fig. 3, Figs S4, S5 and S8; Tables S5, S6 and S13). We also tested whether the species coefficients were correlated with cell volume using linear regressions for each temperature level (Tables S1, S9 and S10; Fig. S7).

**Table 1:** Linear models estimating the effect of temperature, species richness and species composition on ecosystem production. The linear models describe the effect of temperature (T, as a factor), species richness (log_2_(R)), and their interaction on total chlorophyll *a* content of the community (index of production). At each step, terms are added to the linear model and the residual degrees of freedom (res. d.f.) and sum of squares (res. SS) are recalculated. The treatment degrees of freedom (Treat. d.f), sum of squares (treat. SS) and F‐statistic (F) are calculated at each step only for the term that has been added to the model during that step. R^2^ and AIC are calculated for each model. Lower AIC values indicate an improved model. Analyses revealed that the best fitting model included the interaction between temperature and species richness and it explained 40 % of the variance. See Table S2 for a post hoc, multiple comparisons analysis on the slope of the biodiversity–ecosystem function relationship by temperature and Fig. 2 for a graphic representation of the results

Step	Model	Res. d.f.	Res. SS	Treat. d.f.	Treat. SS	*F*	*R* ^2^	AIC
0	Intercept	1394	32 294.1					8345.9
1	step0 + T	1392	23 798.3	2	8495.8	248.5	0.26	7924.1
2	step1 + log_2_(R)	1391	19 886.6	1	3911.7	273.6	0.38	7675.6
3	step2 + T × log_2_(R)	1389	19 345.8	2	540.9	19.4	0.40	7641.1

At the end of the experiment, we estimated the relative abundance of each species within the community from the flow cytometry data using a random forest analysis (see Supplementary Methods). To further explore our hypothesis that variability in species' thermal tolerance plays an important role in mediating the interactive effects of warming and biodiversity loss on ecosystem functioning, we used these data to test whether species abundance in polyculture was correlated with their respective abundance in monoculture using linear regressions of abundance in polyculture as a function of abundance in monoculture for each temperature treatment (Fig. 4). We also quantified whether abundance in monoculture was correlated with species' thermal optima using a linear regression model at each temperature level (Tables S7 and S8; Fig. 3, Figs S4 and S6).

Finally, we estimated net and transgressive overyielding (Tables S12 and S13) by comparing the mean ecosystem function value of the 16‐species polyculture to the mean value of all of the species grown in monoculture (net overyielding) and to the mean value of the species that achieved the highest biomass in monoculture (transgressive overyielding; Cardinale *et al*. 2007).

## Results

**Figure 2:**
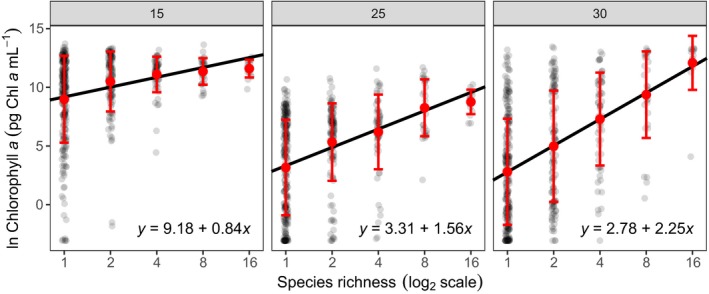
Impact of species loss and warming on ecosystem production. Ecosystem production was quantified as the total chlorophyll *a* content of the community. Grey points correspond to each of the 1395 replicates (*n* = 465 for each temperature treatment). Red point and bars are the mean ± SD for each level of species richness. Lines correspond to the fitted curves from the most parsimonious linear model (see Table 1), with the associated coefficients for each temperature. Post hoc analyses reveal that the slope of the richness‐ecosystem function relationship increased significantly with warming (Table S2), indicating that the impact of the species loss on ecosystem production was more pronounced at higher temperatures.

We found that ecosystem production, measured as total chlorophyll *a*, increased linearly with species richness on a log‐scale, implying a decelerating relationship (Fig. 2). The intercept of the richness–production relationship declined sharply with warming (Fig. 2). Conversely, experimental warming significantly increased the slope of the relationship between richness and ecosystem production, with more than a two‐fold increase (Fig. 2; Table 1, Table S2). The same relationship between the slope of the biodiversity–ecosystem function relationship and temperature was found when using total cell abundance as a proxy for ecosystem production (Fig. S1; Tables S3 and S4).

**Figure 3:**
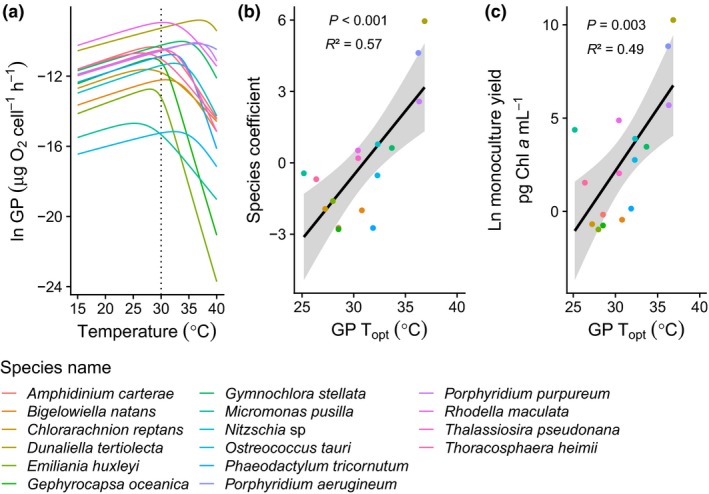
Linking thermal performance traits and species' contribution to community functioning. (a) Thermal performance curves for gross photosynthesis for each species (see Table S14 for parameters and Fig. S8 for detailed fits for each species). (b) Correlation between species coefficient at 30 °C and thermal optimum for gross photosynthesis. Species coefficients represent the contribution of each species to the community functioning and are calculated from the residuals of the random partitions analysis of the diversity–functioning relationships for chlorophyll *a* (Fig. S2). Positive species coefficients indicate species that have a higher than average contribution to ecosystem production, negative coefficients represent lower than average contributions. (c) Correlation between mean yield in monoculture at 30 °C (ln pg Chl *a* mL^−1^) and thermal optimum for gross photosynthesis. Analyses reveal that the thermal optimum for gross photosynthesis was strongly correlated with relative contribution of each species to ecosystem production at 30 °C (Table S5; Fig. S5a) as well as to the yield of each species in monoculture at 30 °C (Table S7; Fig. S6a).

We quantified the contribution of each species present in the community to ecosystem production using the linear model method from Bell *et al*. (2009), which yields a coefficient for each species, where values >0 indicate an above average effect and those <0 are indicative of a below average contribution to production (Figs S2 and S3). We found a positive correlation between species' contributions to community functioning at 30 °C and their thermal optimum of photosynthesis (Figs 3a,b; Table S5), while there was no correlation at 15 and 25 °C (Fig. S5a; Table S5). Similar relationships were found when using total cell abundance instead of chlorophyll *a* to calculate species coefficients (Fig. S4a,b, Fig. S5b; Table S6). We also found that thermal optima of photosynthesis were positively linked to yield in monoculture at 30 °C (Fig. 3c, Figs S4c and S6; Tables S7 and S8). We investigated potential links between cell volume and species' relative contribution to ecosystem production and found no significant relationship at any temperature (Tables S9 and S10; Fig. S7).

**Figure 4:**
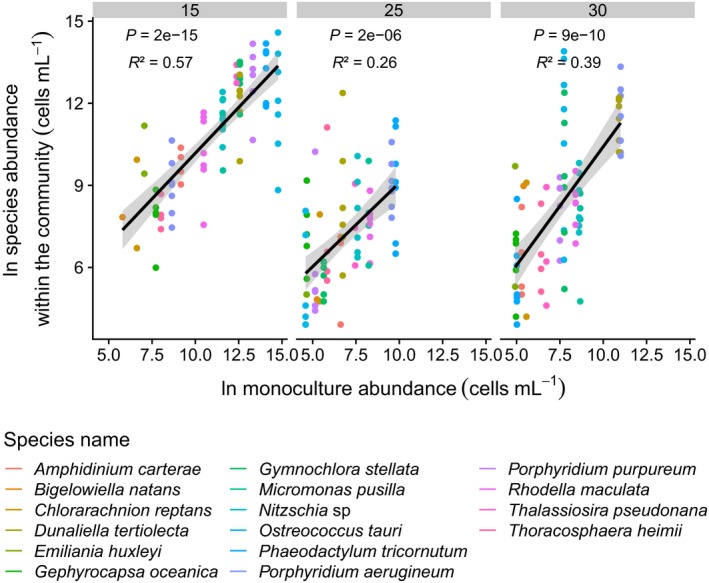
Relationship between focal species abundance in polyculture and its abundance in monoculture for each temperature treatment. Global relationship across all species. Focal species abundance in polyculture is obtained with a randomforest algorithm allowing to assign each cell from a polyculture to its putative species identity (see Supplementary Methods). Because the predictive power of the randomforest algorithm varied with community identity, not all communities were present. We calculated an average abundance of the focal species within the community as the mean of the abundances for the three biological replicates, and an average abundance of the focal species in monoculture as the mean of the biological replicates. There was a positive relationship between focal species abundance within the community and in monoculture (Table S11).

At the end of the experiment, we estimated the relative abundance of each species in the communities. We found that the abundance of each species in polyculture was positively correlated with their abundance in monoculture at all temperature levels (Fig. 4; Table S11). Finally, we estimated net and transgressive overyielding by comparing ecosystem production between the 16‐species polycultures and either the average production of all monocultures (net overyielding) or the production of the best performing species in monoculture (transgressive overyielding). While there was net overyielding at all temperatures, we found no evidence of transgressive overyielding in the 16‐species polycultures compared to the monocultures for ecosystem production measured as chlorophyll *a* content (Table S12). It is worth noting that while results using total cell abundance as a proxy for ecosystem production were largely congruent (Table S13), with some net overyielding at all temperatures, we found some evidence for transgressive overyielding at high temperatures that was not present using chlorophyll *a* content.

## Discussion

By manipulating the species richness and environmental temperature of marine phytoplankton communities in experimental microcosms we found that declines in ecosystem production were far more pronounced under warmer temperatures – i.e. warming led to a steeper relationship between biodiversity and ecosystem functioning. This key result was explicable from an understanding of variability among the phytoplankton taxa in the thermal tolerance of their photosynthetic machinery, with those taxa that had higher thermal tolerance also those which made the largest contributions to ecosystem production in warmer environments.

Ecosystem production increased with species richness and was well characterised by a linear relationship on a log‐scale, indicating that production increased rapidly at low levels of species richness but then decelerated as more species were added to the communities. The intercept of the richness–production relationship, which is indicative of ecosystem production at low levels of richness, declined with warming. This effect of temperature on community biomass is consistent with expectations from metabolic scaling theory and is related to the exponential effects of rising temperature on metabolic rates. When resource availability is fixed and independent of temperature (as was the case in these microcosms), increases in temperature should result in lower equilibrium biomass because each individual uses resources at a faster rate and thus the ecosystem can support fewer individuals (Savage *et al*. 2004).

The steepness of the slope of the relationship between richness and ecosystem production provides a means to assess the importance of diversity for maintaining ecosystem functioning – where a steep slope implies that species loss will have a more marked impact on ecosystem functioning (Reich *et al*. 2012). Consistent with our hypothesis, we found that experimental warming significantly increased the slope of the relationship between richness and production. Thus, as temperatures rose, more species were required to maintain ecosystem functioning at levels comparable with the control. Indeed, only when all 16 species were present were levels of production in the treatment warmed to 30 °C comparable to those at the control temperature. These findings are consistent with recent work on freshwater bacteria, which found that as temperatures depart from ambient conditions (either via warming or cooling) functional redundancy rapidly decays leading to steeper, less saturating diversity–production relationships (García *et al*. 2018).

The steeper relationship between biodiversity and ecosystem functioning in the warmed treatments implies that variance in thermal performance traits might have played an important role in shaping the effects of warming and species loss on ecosystem production. To investigate this, we quantified the contribution of each species present in the community to ecosystem production (Bell *et al*. 2009). We then assessed whether coefficients quantifying the impact of each species in the community on production under the severe warming treatment (30 °C) were correlated with their optimal temperatures for photosynthesis. Here we treat the thermal optimum for photosynthesis as a ‘trait’ that is indicative of variability in thermal performance among the phytoplankton species – i.e. species with higher photosynthetic thermal optima are anticipated to perform better at high temperature than those with low thermal optima. Our analyses do not assume a direct, causal relationship between photosynthetic performance and ecosystem functioning, rather we assume that the thermal optimum for photosynthesis provides useful proxy for differentiating thermal tolerance among the 16 species of marine phytoplankton. Indeed recent work has shown that photosynthetic performance is a key trait determining competitive fitness in phytoplankton (Schaum *et al*. 2017). We found a highly significant positive correlation between species' contribution to ecosystem production and their thermal optimum for gross photosynthesis in the high temperature treatment, indicating that those species which contributed positively to ecosystem function under severe warming were also those with the highest thermal tolerance of their photosynthetic machinery. We further found the same association between yield in monoculture under severe warming and thermal tolerance. Moreover, species performance within a community was positively associated with its performance in monoculture. Together, this shows that the relative contribution of each species to ecosystem production in the warm treatment was strongly dependent on the thermal tolerance of their photosynthetic machinery and their performance in monoculture. In warmer conditions, communities with low species richness have a lower probability of including those species with high thermal tolerance that can contribute positively to ecosystem function.

Another important driver of metabolism, and consequently community structure and ecosystem function in phytoplankton communities, is cell size (Marañón 2015). Cell size is a key trait for understanding phytoplankton nutrient uptake (Marañón 2015), and recent work has emphasised the key role of nutrient physiology traits can play in mediating phytoplankton responses to climate change (Thomas *et al*. 2017; Bestion *et al*. 2018b). For instance, variability in marine phytoplankton growth rate across latitudes has been shown to be strongly linked to nutrient availability (Marañón *et al*. 2014), while the contribution of the smallest‐sized phytoplankton cells to total phytoplankton biomass in the ocean has been shown to increase with temperature (Morán *et al*. 2010). Cell volume has also been found to correlate with the optimum growth temperature in marine phytoplankton with smaller cells typically able to tolerate higher temperatures (Sal *et al*. 2015; Barton & Yvon‐Durocher 2019). We therefore investigated potential links between species' contribution to ecosystem production and cell size. We found no significant association at any of the temperature treatments. This result suggests that changes in the biodiversity–ecosystem function relationship were not related to size dependent turnover in species composition. In general, our results show that ecosystem production in the warm environment was strongly dependent on the presence of species with high photosynthetic thermal optima to maintain ecosystem function. Thus, when biodiversity loss removed these species and their associated traits from the community, the negative impact on ecosystem functioning was marked, as evidence by the steep richness‐production slope in the warm treatments.

We estimated the net overyielding (i.e. the difference between the mean ecosystem function of the 16‐species polyculture and the mean ecosystem function of the monocultures) and transgressive overyielding (i.e. the difference between the mean ecosystem function of the 16‐species polyculture and the ecosystem function of the best functioning monoculture) (Cardinale *et al*. 2007). This allowed us to investigate overyielding due to both selection and complementarity effects from overyielding only due to complementarity effects. We found that community performance was only ever as good as the best species in monoculture implying little evidence for transgressive overyielding in any of the 16‐species polycultures when using chlorophyll *a* as a proxy for biomass. This suggests that selection effects played an important role in mediating changes in the relationship between biodiversity and ecosystem production across the temperature gradient. However, we did see some evidence for transgressive overyielding at the highest temperature only when using cell abundance as a proxy. Such transgressive overyielding could be driven by the coexistence of diverse size classes of algae, each with different pigment characteristics related to their size, which may have led to discrepancies between calculations based on total abundance and total chlorophyll *a*. Taken together, these results suggest that the loss of phytoplankton species from planktonic communities might have a much more pronounced negative impact on marine primary production in a warmer world.

It is important to consider that our findings might be impacted by the choice of phytoplankton species used in this study. Because both temperate and tropical species were used in our experiments (Table S1), the species pool encompassed a wide range of thermal optima. In the ocean, the regional species pool for a given location might be expected to display a narrower range of thermal traits if long‐range dispersal is limited. Low variance of thermal tolerance traits would be expected to lead to a less pronounced impact of temperature change on the slope of the diversity–production relationship but a more pronounced collapse of ecosystem function when warming exceeds the upper thermal tolerance in the regional species pool. Nevertheless, recent work has demonstrated that minimum connectivity times between even the most distant ocean basins are on the order of a decade (Jönsson & Watson 2016), which is likely to lead to mixing of temperate and tropical taxa over timescales relevant to climate warming. Furthermore, planktonic microorganisms possess an enormous potential for dispersal, allowing for reshuffling of communities (Finlay 2002). The range of thermal optima among the species in our study (11.7 °C) corresponds closely to the range observed in marine phytoplankton isolated from one tenth of a degree of latitude in the ocean (10.7 °C, see fig. 1 from Thomas *et al*. 2012 and Table S15). Thus, even though the species used in our study originated from diverse latitudes, it is likely that the variance in thermal optima is consistent with thermal trait variation at local to regional scales in the ocean. Another important caveat is that our experiments were carried out in microcosms, which might influence the broader applicability of our results. Microcosm environments lack the complexity and heterogeneity of the natural environment and do not allow species to partition their niches along the full diversity of environmental axes that may be possible in nature. Thus, it is likely that more complex biotic and abiotic environments could lead to more niche partitioning and/or complementarity among the phytoplankton species. For instance, Burgmer & Hillebrand (2011) found that the presence of consumers modulated the effect of temperature on both algal biomass and species richness, switching the impacts of warming on the species richness and biomass from negative to positive in the presence of grazers. Nevertheless, it is important to recognise that our aim with this work was not to replicate the complexity of the natural environment, but rather our principal objective with these experiments were to unpick the mechanisms that determine how changes in temperature influence the relationship between phytoplankton diversity and ecosystem production. Clearly, further work in more complex environmental settings is required to translate these findings into natural settings.

Our findings highlight the potential for major synergistic negative impacts of species loss and environmental warming on the production of marine phytoplankton communities. We found that the slope of the relationship between species richness and ecosystem production increased significantly as temperatures rose above ambient conditions. Consequently, ecosystem production declined much more abruptly as species were lost from the communities in the warmer treatments and therefore a greater number of species were required to maintain ecosystem functioning at levels comparable with the control. This pattern was linked to variance in thermal traits in the species pool. When temperatures exceeded the optimum for some species but not others, communities with low species richness had a reduced probability of including taxa with thermal traits that enabled them to maintain high levels of production in the warm environment and experienced dramatic declines in ecosystem functioning. Overall, these results suggest that if biodiversity loss of marine phytoplankton is not correlated with thermal performance traits, warming could lead to a marked negative impact of species loss on ecosystem production. This could conceivably occur if other stressors which result in the loss of phytoplankton species from communities – such as invasive species, nutrient limitation, pollution, acidification, top‐down control – are decoupled in time and/or space from ocean warming (Suchanek 1994; Monaco & Prouzet 2015). Indeed, major changes in food web structure due to overharvesting and changes in top‐down control are known to be a key driver of biodiversity loss in marine ecosystems (Pauly *et al*. 1998) and are likely to be largely independent of thermal performance traits. However, if biodiversity loss is directly linked to climate warming (Thomas *et al*. 2004; Bestion *et al*. 2017), it should be non‐random relative to thermal performance traits (Thomas *et al*. 2012) and the marked negative impact on production could be buffered to some degree, because the species with lower thermal tolerance that contribute least to production in the new environment (i.e. those with lower species coefficients, see Fig. 3), will be the first to disappear. Overall, our results provide the first empirical evidence of the critical role that species‐ and thermal trait diversity could play in mediating the effects global warming on the primary production of marine phytoplankton.

## Author Contributions

EB and GYD conceived the study. EB designed the experiment, EB, SB, FG and RW performed the experiment. EB and SB analysed the data. EB and GYD wrote the manuscript and all authors contributed to revisions. The authors declare no competing interests.

## Data Availability

Data are available on zenodo at https://doi.org/10.5281/zenodo.3555223.
