# Supplementary material for: Abrupt declines in marine phytoplankton production driven by warming and biodiversity loss in a microcosm experiment
Source: Ecol Lett. 2020 Jan 11;23(3):457–66. doi: 10.1111/ele.13444 (PMC7007813; doi:10.1111/ele.13444)
Supplement: Supplementary file 1 [file ELE-23-457-s001.pdf]

Supplementary Material For

Abrupt declines marine phytoplankton production driven by warming and biodiversity loss in a microcosm experiment

Elvire Bestion, Samuel Barton, Francisca C. García, Ruth Warfield, Gabriel Yvon-Durocher

Ecology Letters, doi: 10.1111/ele.13444

Contents

Supplementary Methods 3

Species and culture conditions 3

Thermal performance assays 3

Biodiversity-function experiment 5

Chlorophyll *a* calibration 5

Data analyses 6

Supplementary References 7

Supplementary figures 9

Fig. S1: Biodiversity-ecosystem functioning relationship across temperatures for the abundance proxy. 9

Fig. S2: Species coefficients for chlorophyll a by temperature. 10

Fig. S3: Species coefficients for abundance by temperature. 11

Fig. S4: Link between thermal performance and species contribution to community functioning from the community abundance proxy. 12

Fig. S5: Link between thermal performance and species relative contribution to community functioning at each of the three temperature levels 13

Fig. S6: Link between thermal performance and species yield in monoculture at each of the three temperature levels 14

Fig. S7: Link between cell volume and species relative contribution to community functioning at each of the three temperature levels 15

Fig. S8: Thermal performance curves for gross photosynthesis for each of the 16 phytoplankton species. 16

Supplementary tables 17

Table S1: Detailed information about the species 17

Table S2: Contrast analysis of the slopes of the biodiversity-ecosystem functioning relationship 18

Table S3: Linear models estimating the effect of temperature, species richness and species composition on ecosystem production measured as community abundance. 19

Table S4: Contrast analysis of the slopes of the biodiversity-ecosystem function relationship ... 19
Table S5: Impact of Topt for gross photosynthesis on species coefficient for chlorophyll a by temperature ... 20
Table S6: Impact of Topt for gross photosynthesis on species coefficient for community abundance by temperature ... 20
Table S7: Impact of Topt for gross photosynthesis on species yield in monoculture (as chlorophyll a) by temperature ... 21
Table S8: Impact of Topt for gross photosynthesis on species yield in monoculture (as abundance) by temperature ... 21
Table S9: Impact of cell volume on species coefficient for chlorophyll *a* by temperature ... 22
Table S10: Impact of cell volume on species coefficient for abundance by temperature ... 22
Table S11: Relationship between focal species abundance in polyculture and its abundance in monoculture by temperature ... 23
Table S12: Net and transgressive overyielding for chlorophyll a. ... 24
Table S13: Net and transgressive overyielding for abundance ... 24
Table S14: Parameters of the thermal performance curves ... 25
Table S15: Spread of optimum values compared to natural spreads in optimum values locally ... 26

Supplementary Methods

Species and culture conditions

The experiment was conducted with 16 species of marine phytoplankton, *Amphidinium carterae, Bigelowiella natans, Chlorarachnion reptans, Dunaliella tertiolecta, Emiliania huxleyi, Gephyrocapsa oceanica, Gymnochlora stellata, Micromonas pusilla, Nitzschia sp., Ostreococcus tauri, Porphyridium aerugineum, Porphyridium purpureum, Phaeodactylum tricornutum, Rhodella maculata, Thoracosphaera heimii,* and *Thalassiosira pseudonana* (Table S1). Strains of each species were ordered from the Culture Collection of Algae and Protozoa (www.ccap.ac.uk) and RCC (Roscoff Culture Collection). These strains varied widely in their geographic provenance, from the North Atlantic (most strains) to the Mediterranean Sea and the West and South Pacific (Table S1). Stocks of each of the strains were cultured on their previous culture collection mediums using artificial seawater prior to the experiment and during the thermal performance assays. We used the following media: Guillard's F/2 and F/2 + Si, Keller's K, K + Si and K/2 (Table S1). Species were maintained in semi-continuous culture in an Infors-HT shaking incubator (65 rpm) at 20°C on a 12:12 light-dark cycle with a light intensity of 45-50 $\mu mol \cdot m^{-2} \cdot s^{-1}$. Cultures were kept under exponential, nutrient replete, growth conditions for ~ 2 months before any physiological data was collected. Prior to the community experiment, species were acclimated to a new culture medium, the K + Si culture medium, for one month until balanced growth in semi-continuous culture was achieved. This was done in order to use a medium common to all species for the community experiment, as it would not have been possible to mix species from different media. We chose the K+ Si culture medium as a medium for the community experiment as it was the more complete medium, containing all of the chemical components present in the F/2 + Si medium apart from the sodium phosphate monobasic, switched to sodium beta glycerophospate, and the cobalt chloride, switched to cobalt sulfate, and containing several other elements (i.e. ammonium chloride, selenous acid, Tris base and FeNaEDTA). This ensured that no species would struggle to grow from a lack of specific nutrients. Allowing one month of acclimation allowed verifying that the growth of the species in the new medium was roughly similar to their growth in their original medium. It is possible that the growth would be slightly different when changing growth medium, however, it is unlikely that it would affect the thermal optimum of the species, as these media are not limiting for the key nutrients required by the marine phytoplankton species in this study.

Thermal performance assays

We characterised acute thermal performance curves for gross photosynthesis for each of the 16 species (see Fig. 1 for a detailed flow diagram of the experiment). Acute thermal performance curves are immediate responses to temperature, and might differ from thermal performance measured after a period of acclimation to novel temperatures. However, such acute thermal performances have been related to phytoplankton fitness as measured by population growth rate (Padfield *et al.* 2016), and to competitive ability of phytoplankton (Schaum *et al.* 2017). Photosynthesis and respiration measurements from 7 to 49°C where made for a minimum of 3 biological replicates per species. We used a clark-type oxygen electrode as part of a Chlorolab 2 system (Hansatech Ltd, King's Lynn, UK) to measure net rates of oxygen evolution in the light (net primary production, NP) and oxygen consumption in the dark (dark respiration); both in units of $\mu mol\ O_2\ mL^{-1}\ s^{-1}$. All biological replicates were sampled from the stock cultures, which had all been growing at 20°C and were taken at the mid-logarithmic growth phase to ensure that the samples were not nutrient limited. To improve the signal to noise ratio when measuring rates, all biological replicates were concentrated by centrifugation at 1500 rpm, 20°C for 15 minutes and re-suspended into an adequate volume of growth medium. This centrifugation technique is commonly used in studies measuring thermal performance curves of gross photosynthesis (Padfield *et al.* 2016; Schaum *et al.* 2017; Barton & Yvon-Durocher 2019), however it is to note that it might potentially affect performance due to density effects. Prior to running a sample at each assay temperature, all samples were given ~ 15 minutes to pre-acclimate to the assay temperature in the dark before any data was collected. This was necessary for two reasons, i) as the sample adjusts to the assay temperature this will naturally cause changes in the dissolved oxygen concentration, ii) the electrode system results in oxygen signal drift, and this too is temperature dependent. We measured

rates of oxygen depletion from 21 sterilised artificial seawater samples across a range of temperatures 4°C - 44°C and found that the impact of drift was minimised after ~15 minutes of stabilisation time. Nevertheless, signal drift was linearly temperature dependent after this time. To account for drift in our dataset we corrected all our raw data using the following empirically derived relationship:

$$drift = (-0.392 \times T) - 6.51 \quad (1)$$

Where *T* is assay temperature (°C) and *drift* is the non-biological depletion in oxygen concentration measured in units µmolO_2 mL^{-1} s^{-1} after approximately 15 minutes of stabilisation. The raw O_2 flux data was then corrected by subtracting the estimated drift.

Rates of net photosynthesis, measured as O_2 evolution, were collected across a range of light intensities from 0 to 1800 µmol m^2 s^{-1} with increments of 50 µmol m^2 s^{-1} between 0 to 200 µmol m^2 s^{-1}, 100 µmol m^2 s^{-1} between 200 and 1000 µmol m^2 s^{-1}, followed by 1200 µmol m^2 s^{-1}, 1500 µmol m^2 s^{-1} and finally 1800 µmol m^2 s^{-1}. This enabled us to model a photosynthesis-irradiance (PI) curve for each assay temperature, and therefore obtain an estimate of photosynthesis, NP_{max}, where the rate was not limited by light intensity (see equation 2). Respiration (R) was measured in the dark, as oxygen consumption, over a 3-minute period directly following the light response outlined above.

The PI curves for NP were fitted to the following model for photoinhibiton (Eilers & Peeters 1988; Edwards *et al.* 2016):

$$NP(I) = \frac{NP_{max}I}{\frac{NP_{max}}{\alpha I_{opt}^2}I^2 + \left(1 - 2\frac{NP_{max}}{\alpha I_{opt}}\right)I + \frac{NP_{max}}{\alpha}} \quad (2)$$

Where $NP(I)$ is the rate of net primary production at light intensity, I, NP_{max} is the maximum rate of NP at the optimal light intensity, I_{opt}, α is the rate in which NP increases up to NP_{max}. Models were fitted using the nlsLoop function in the R github package nlsLoop (https://github.com/padpadpadpad/nlsLoop). This draws on the nlsLM function in the minpack.lm R package, which uses a modified Levenberg-Marquardt optimisation algorithm. Model parameters were determined by running 2000 random combinations of estimated starting parameters, which were then selected using the Akaike Information Criterion (AIC) to determine the set of parameters that best characterised the data based on equation 2 (Padfield *et al.* 2017).

Light saturated gross primary production (GP) was then calculated for each assay temperature as:

$$GP = NP_{max} + R \quad (3)$$

Metabolic rates were then converted from units µmol O_2 mL^{-1} s^{-1} to µg O_2 $cell^{-1}$ $hour^{-1}$ by multiplying the rate by the molecular weight of O_2, 32, and dividing by the number of cells mL^{-1} measured for each biological replicate using flow cytometry.

We quantified the temperature-dependence of gross photosynthesis rates using the modified Sharpe-Schoolfield equation (Sharpe & DeMichele 1977; Schoolfield *et al.* 1981):

$$\ln\big(b(T)\big) = E_a\left(\frac{1}{kT_c} - \frac{1}{kT}\right) + \ln\big(b(T_c)\big) - \ln\left(1 + e^{E_h\left(\frac{1}{kT_h} - \frac{1}{kT}\right)}\right) \quad (4)$$

where *b* is the metabolic rate (GP in µgO_2 $cell^{-1}$ $hour^{-1}$), *k* is Boltzmann's constant (8.62×10^{-5} eV K^{-1}), E_a is the activation energy (eV), indicative of the steepness of the slope leading up to the thermal optima, T is temperature in Kelvin (K), E_h is the deactivation energy which characterizes temperature-induced decrease in rates above T_h where half the enzymes have become non-functional and $b(T_c)$ is rate normalized to an arbitrary reference temperature, here T_c = 20ºC (+ 273.15), where no low or high temperature inactivation is experienced. We fitted the data for gross photosynthesis across all species to Eq. 4 using non-linear mixed effects modelling with the nlme package in R. The models included each of the parameters in Eq. 4 as fixed effects, which quantify the average value of the parameter

across all species and replicates. We also included 'replicate' nested within 'species' to account for the fact that we measured a minimum 3 replicate thermal response curves for each species. Here the random effect quantifies species-specific deviations from the fixed effects as well as those attributable to variance among the replicates of each species.

Finally, by differentiating Eq. 4 and solving for the global maxima an optimum temperature can be estimated using the following expression

$$T_{opt} = \frac{E_h T_h}{E_h + kT_h \ln(\frac{E_h}{E_a} - 1)} \quad (5)$$

Biodiversity-function experiment

Artificial communities for the biodiversity-functioning experiment were designed using the random partition design described by Bell *et al.* (2009). We randomly divided species into communities with increasing species richness levels from 1, 2, 4, 8, and 16 species, where for each species richness level, the community assemblages were constructed by sampling all of the 16 species without replacement (Fig. 1). This allowed each species to be represented an equal number of times at each richness level. This process was repeated to form 5 independent partitions of the species pool, so that for each richness level (R) the number of assemblages was 5 x 16/R. Each assemblage was then replicated 3 times. Further, all replicated communities were subjected to three temperature treatments, 15, 25 and 30°C, giving for the experiment as a whole a total of 3 x 3 x 5 x (16 + 8 + 4 + 2 + 1) = 1395 communities.

The biodiversity-function experiment was done in sixty 24 well plates filled with 2 mL of K+Si medium (20 plates per temperature). Each well was inoculated with 1600 cells.mL^{-1} of each community (i.e. 100 cells.mL^{-1} per species in the case of sixteen-species communities, 200 cells.mL^{-1} per species for eight-species communities, 400 cells.mL^{-1} per species for four-species communities, 800 cells.mL^{-1} per species for two-species communities, and 1600 cells.mL^{-1} per species for monocultures). The position of the communities was randomised within the plates.

Plates were covered with AeraSeal breathable membrane, minimising evaporation and contamination but allowing gas exchange. Samples were then grown in three Infors-HT shaking incubators at 15, 25 and 30°C on a 12:12 light cycle. Every 5 days, we added 0.5, 1, and 1.2 ml of distilled water to each well of the 15, 25 and 30°C plates respectively to refill water loss due to evaporation, and the position of the plates was randomised within the incubator. After 19 days of the experiment, we took a 100 µL sample from each community on a 96 well plate. We preserved the samples with 10 µL of 1% sorbitol solution as a cryoprotectant. After one hour of incubation in the dark, samples were frozen at -80°C until further analysis. Plates were thawed in a water bath at ca. 38°C for 10 minutes and cell density in each sample was determined by flow cytometry (BD Accuri C6). Plates were run on the flow cytometer on slow flux settings (14 µL·min-1), counting 20 µL of each sample. Cleaning fluid was run after each sample to avoid contamination of measurements between samples.

Chlorophyll *a* calibration

We grew each species separately, and when in exponential phase we calculated cell density and mean FL3 values on the flow cytometer as above. We then centrifuged a 50 mL sample at 3500 rpm for 30 minutes at 4°C. The resultant pellets were re-suspended in 6 mL of ethanol (100%), and all samples were then kept refrigerated in the dark for 24 hours. Following the extraction period, samples were vortexed and we measured absorbance from 610 nm to 750 nm for a minimum of three technical replicates per species using a spectrophotometer (Jenway 7315). Blanks were measured across the same wavelength range to correct for the ethanol absorbance. We then used well established absorbance coefficients to obtain estimates of chlorophyll *a* cell^{-1} for the exponentially growing cultures (Ritchie 2006; Henriques *et al.* 2007). We modelled the chlorophyll *a* values in picograms against FL3 values from the flow cytometer with a linear model on a log-log scale (linear model, estimates (± SE): intercept = -16.46 ± 2.18, slope: 1.18 ± 0.16, t = 7.41, p = 3e^{-6}, R^2 = 0.78). This calibration curve was then used to estimate Chl *a* content of cultures from FL3 values from the flow cytometer. We did not do a calibration of the chlorophyll *a* transformation per temperature treatment. This could potentially affect

the results if the calibrations vary between temperatures. However, it is to note that re-analysing the results without calibration by using the raw FL3 values from the flow cytometer leads to very similar results (best model investigating the relationship between temperature and biodiversity-ecosystem functioning: ln(FL3 fluorescence) ~ T*$\log_2$(R), F = 18.4, R^2 = 0.40, ΔAIC = 32.6 compared to F = 19.4, R^2 = 0.40 and ΔAIC = 34.5 for the same model with calibrated data (Table 1); slope of the diversity-production relationship : 0.81 ± 0.15, 1.44 ± 0.15 and 2.10 ± 0.15 respectively for 15°C, 25°C and 30°C for the fluorescence data compared to 0.84 ± 0.16, 1.56 ± 0.16 and 2.25 ± 0.16 at the three same temperatures for the calibrated data). Further, analysing data with cell abundance leads to very similar results than with transformed chlorophyll *a* (see comparison between results in Table S3, S4 and in Table 1, S2), suggesting that potential deviations in the calibration of the chlorophyll *a* transformations are not an issue.

Data analyses

All statistical analyses were undertaken using R v3.4.2 (R Core Team 2014). Cell counts and the associated cytometric properties (forward scatter (FSC, a proxy of size), side scatter (SSC), red fluorescence (FL3, a proxy of chlorophyll *a* content)), green fluorescence (FL1), orange fluorescence (FL2) and blue fluorescence (FL4) were stored in flow cytometry standard (FSC) files created by the flow cytometer. The FCS files were read into R using the Bioconductor package FlowCore. We first filtered the data to remove noise by removing every data point where either $\log_{10}$(FSC)<5, $\log_{10}$(SSC)<5 and/or $\log_{10}$(FL3)<3.5, which are below minimum values observed for live cells of these species. We then used FL3 values to derive cell chlorophyll *a* content in picogram per cell using the calibration curve described above.

We calculated community abundance as the total number of cells, and total chlorophyll *a* content as the sum of chlorophyll *a* across all cells scaled to numbers per mL. These two metrics were then used as proxies of ecosystem production, as found in other studies (Boyd *et al.* 2013). We focus on chlorophyll *a* content in the main text, as it is the most widely proxy for studying phytoplankton biomass (Field *et al.* 1998; Marañón *et al.* 2014), but we also showed that the results are mostly consistent when using community abundance in supplementary material (Table S3-S4, S6, S8, S10, S12, Fig. S1, S3, S4, S5b, S6b, S7b).

The biodiversity and ecosystem functioning relationship was analysed using the analysis of variance method described by Bell *et al.* (2009). Factors relating to temperature treatments were fitted first, followed by log-transformed species richness and their interaction. The best model always included the temperature by richness interaction (see Table 1, S3 respectively for ecosystem functioning measured as chlorophyll *a* or community abundance). We used post-hoc contrasts to assess whether the slope of the biodiversity-ecosystem function relationship differed between each pairwise combination of temperature levels with the lstrends function from lsmeans package with tukey method of p-value adjustment (Table S2, S4). We then extracted the residuals from a model of ecosystem functioning by log richness for each temperature treatment and fitted these residuals to the presence-absence status of each of the 16 species. The species coefficients provided by this method give a measure of the effect of each species on ecosystem production relative to an average species, where positive values indicate an above average contribution and negative values a below average contribution (Fig. S2-S3). We then linked these species coefficients to each species' thermal optimum for photosynthesis (Table S13, Fig. S8) through linear models, separating data by each temperature treatment (Table S5-S6, Fig. 3a,b, S4a,b, S5). We tested through linear models separating data by temperature treatment whether these species coefficients depended on another functional trait, namely cell volume (in μm^3, see Table S1, Table S9-S10) measured as part of another study (Barton & Yvon-Durocher 2019). Indeed, cell size can be used as a proxy to control for nutrient competitive ability of the species (Marañón 2015), and can be linked to species responses to a warming ocean (Sal *et al.* 2015).

We then aimed to distinguish which species were actually present in the communities at the end of the experiment, and their abundance within the communities. We used the flow cytometry output as values to define species morphology and pigment composition and thus discriminate between species in polycultures with a randomforest analysis. We first separated the monoculture dataset into a training

and a testing dataset. For each pre-existing community composition in the polycultures, we restricted the training and testing dataset to the species originally present inside of the community, and then used a randomforest algorithm on the training dataset using the 10 variables returned by the flow cytometer (that is FSC.H, FSC.A, SSC.H, SSC.A, FL1.H, FL1.A, FL2.H, FL2.A, FL3.H, FL3.A, FL4.H and FL4.A, .H standing for height and .A for area). We then used these discrimination functions to predict community composition from the training dataset, and calculate a percentage of accuracy in the prediction for each species present in the community. Unfortunately, some species were difficult to tease apart; for instance, *Chlorarachnion reptans* presence was consistently poorly detected, except in biculture communities with *Nitzschia sp* or *Phaeodactylum tricornutum*. We therefore decided to use a cut-off of community predictability, where the worst predicted species in the community would be predicted with at least 60 % of accuracy. Such a cut-off led us to exclude 37 out of the 71 communities from our prediction of species abundance, including the 16 species-rich community and all of the 8 species-rich communities. The remaining communities had a mean prediction accuracy (the average of the prediction power of each species inside of the community) of 91.4 % ± 6.6 SD, and a minimum prediction accuracy (the prediction power of the worst predicted species inside of the community) of 85.8 % ± 11.5 SD, which makes us confident in the assessed abundance of each species for these communities. We then applied the discrimination algorithms to the polycultures from the experiment (excluding the polycultures for which the predictive power was too low), and calculated abundance as cell counts for each species. For each temperature treatment, we used a linear model of abundance in polycultures by abundance in monocultures to test whether both abundances were related (Fig. 4, Table S11). We then studied how these mean yields in monoculture were linked to thermal optimum through a linear model of yield as a function of T_{opt} separating the data by temperature treatment (Table 7-S8).

Finally, we estimated net and transgressive overyielding (Table S12-S13) by comparing the mean ecosystem function value of the 16-species polyculture across all replicates to the mean value of all replicates for all species grown in monoculture (net overyielding) and comparing the mean value of the 16-species polyculture to the mean value of all replicates of the species that achieves the highest biomass in monoculture (transgressive overyielding (Cardinale *et al.* 2007)). This allowed us to tease apart overyielding due to both selection and complementarity effects from overyielding only due to complementarity effects.

Supplementary References

Barton, S. & Yvon-Durocher, G. (2019). Quantifying the temperature dependence of growth rate in marine phytoplankton within and across species. *Limnology and Oceanography*, 64, 2081–2091.

Bell, T., Lilley, A.K., Hector, A., Schmid, B., King, L. & Newman, J.A. (2009). A Linear Model Method for Biodiversity–Ecosystem Functioning Experiments. *The American Naturalist*, 174, 836–849.

Boyd, P.W., Rynearson, T.A., Armstrong, E.A., Fu, F., Hayashi, K., Hu, Z., *et al.* (2013). Marine Phytoplankton Temperature versus Growth Responses from Polar to Tropical Waters – Outcome of a Scientific Community-Wide Study. *PLoS ONE*, 8, e63091.

Cardinale, B.J., Wright, J.P., Cadotte, M.W., Carroll, I.T., Hector, A., Srivastava, D.S., *et al.* (2007). Impacts of plant diversity on biomass production increase through time because of species complementarity. *PNAS*, 104, 18123–18128.

Edwards, K.F., Thomas, M.K., Klausmeier, C.A. & Litchman, E. (2016). Phytoplankton growth and the interaction of light and temperature: A synthesis at the species and community level. *Limnol. Oceanogr.*, 61, 1232–1244.

Eilers, P.H.C. & Peeters, J.C.H. (1988). A model for the relationship between light intensity and the rate of photosynthesis in phytoplankton. *Ecological Modelling*, 42, 199–215.

Field, C.B., Behrenfeld, M.J., Randerson, J.T. & Falkowski, P. (1998). Primary Production of the Biosphere: Integrating Terrestrial and Oceanic Components. *Science*, 281, 237–240.

Henriques, M., Silva, A. & Rocha, J. (2007). Extraction and quantification of pigments from a marine microalga: a simple and reproducible method. *Commun. Curr. Res. Educ. Top. Trends Appl. Microbiol.*, 586–593.

Marañón, E. (2015). Cell Size as a Key Determinant of Phytoplankton Metabolism and Community Structure. *Annu. Rev. Mar. Sci.*, 7, 241–264.

Marañón, E., Cermeño, P., Huete-Ortega, M., López-Sandoval, D.C., Mouriño-Carballido, B. & Rodríguez-Ramos, T. (2014). Resource Supply Overrides Temperature as a Controlling Factor of Marine Phytoplankton Growth. *PLoS ONE*, 9, e99312.

Padfield, D., Lowe, C., Buckling, A., Ffrench-Constant, R., Student Research Team, Jennings, S., *et al.* (2017). Metabolic compensation constrains the temperature dependence of gross primary production. *Ecol Lett*, 20, 1250–1260.

Padfield, D., Yvon-Durocher, G., Buckling, A., Jennings, S. & Yvon-Durocher, G. (2016). Rapid evolution of metabolic traits explains thermal adaptation in phytoplankton. *Ecol Lett*, 19, 133–142.

R Core Team. (2014). *R: A Language and Environment for Statistical Computing*. R Foundation for Statistical Computing, Vienna, Austria.

Ritchie, R.J. (2006). Consistent Sets of Spectrophotometric Chlorophyll Equations for Acetone, Methanol and Ethanol Solvents. *Photosynth Res*, 89, 27–41.

Sal, S., Alonso-Sáez, L., Bueno, J., García, F.C. & López-Urrutia, Á. (2015). Thermal adaptation, phylogeny, and the unimodal size scaling of marine phytoplankton growth: Temperature, phylogeny, & phytoplankton allometry. *Limnol. Oceanogr.*, 60, 1212–1221.

Schaum, C.-E., Barton, S., Bestion, E., Buckling, A., Garcia-Carreras, B., Lopez, P., *et al.* (2017). Adaptation of phytoplankton to a decade of experimental warming linked to increased photosynthesis. *Nature Ecology & Evolution*, 1, 0094.

Schoolfield, R.M., Sharpe, P.J.H. & Magnuson, C.E. (1981). Non-linear regression of biological temperature-dependent rate models based on absolute reaction-rate theory. *Journal of Theoretical Biology*, 88, 719–731.

Sharpe, P.J.H. & DeMichele, D.W. (1977). Reaction kinetics of poikilotherm development. *Journal of Theoretical Biology*, 64, 649–670.

Thomas, M.K., Kremer, C.T., Klausmeier, C.A. & Litchman, E. (2012). A Global Pattern of Thermal Adaptation in Marine Phytoplankton. *Science*, 338, 1085–1088.

Supplementary figures

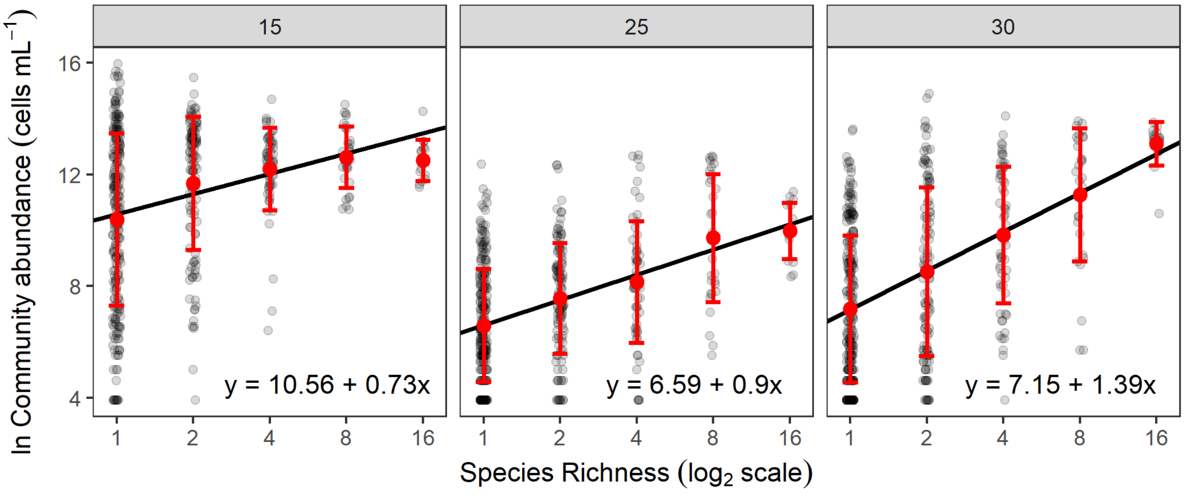

Fig. S1: Biodiversity-ecosystem functioning relationship across temperatures for the abundance proxy. Impact of species richness on ecosystem production measured as community abundance (ln(cells.ml^{-1})) by temperature over 19 days. Points represent each of the 1395 samples (N = 465 by temperature). In red, the mean ± SD for each level of species richness. Lines are from the most parsimonious linear model, that is model 3 in Table S3 ((lm(ln(community abundance) ~ temperature* $\log_2$(Richness))), with the associated coefficients for each temperature. The slope of the richness-ecosystem function relationship increases with temperature (Table S4), suggesting that the impact of the loss of a species on ecosystem production is more important at higher temperatures. The figure is analogous to Fig. 2 from the main document, and shows that the results are congruent regardless of the ecosystem production proxy used.

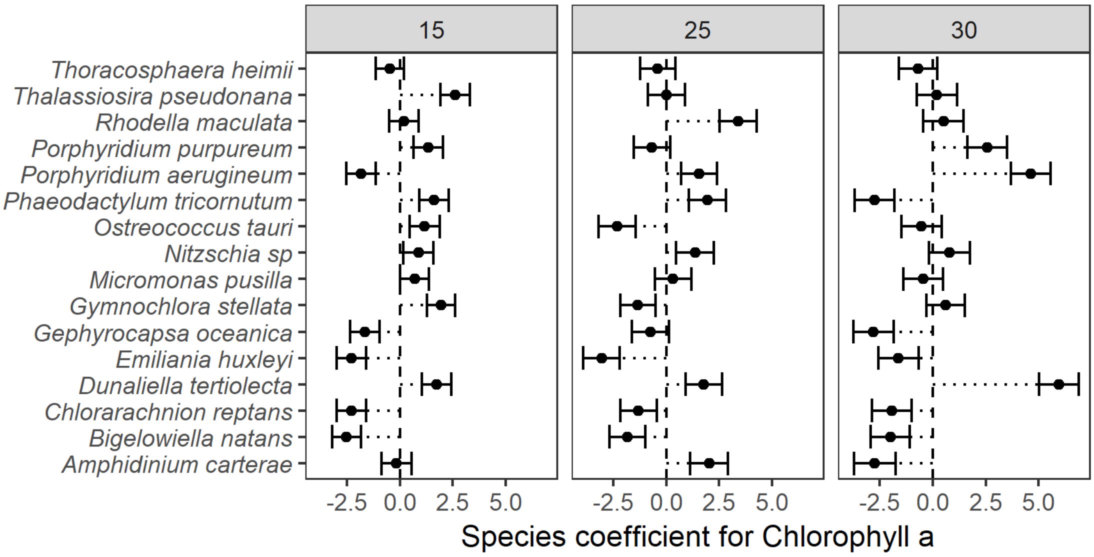

Fig. S2: Species coefficients for chlorophyll a by temperature. Impact of species identity on community functioning after taking into account the impact of species richness. To take into account the impact of species richness, the model uses the residuals of ln(chlorophyll *a*)~$\log_2$(R) for each temperature as a dependent variable. For each temperature, this dependent variable is modelled against each of the 16 species modelled as a bivariate presence-absence variable. The models explain 39 % of the variance (adjusted R^2) at 15°C, 33 % of the variance at 25°C, and 46% of the variance at 30°C. The figure shows the linear model coefficients for each species at each temperature, with the 95% confidence intervals around each coefficient. Different species have different relative contributions to ecosystem functioning, and these contributions vary with temperature. For instance, *Phaeodactylum tricornutum* has a positive relative contribution to ecosystem functioning at low and medium temperatures, meaning that the addition of this species has a stronger positive effect than the addition of an average species, while the relative contribution turns negative at high temperature, meaning that the addition of the species has a smaller positive impact than average. Conversely, the contribution of *Porphyridium aerugineum* turns from negative to positive when temperature increases. Other species' relative contribution does not change with temperature, as for instance in *Bigelowiella natans,* which always has a negative relative contribution.

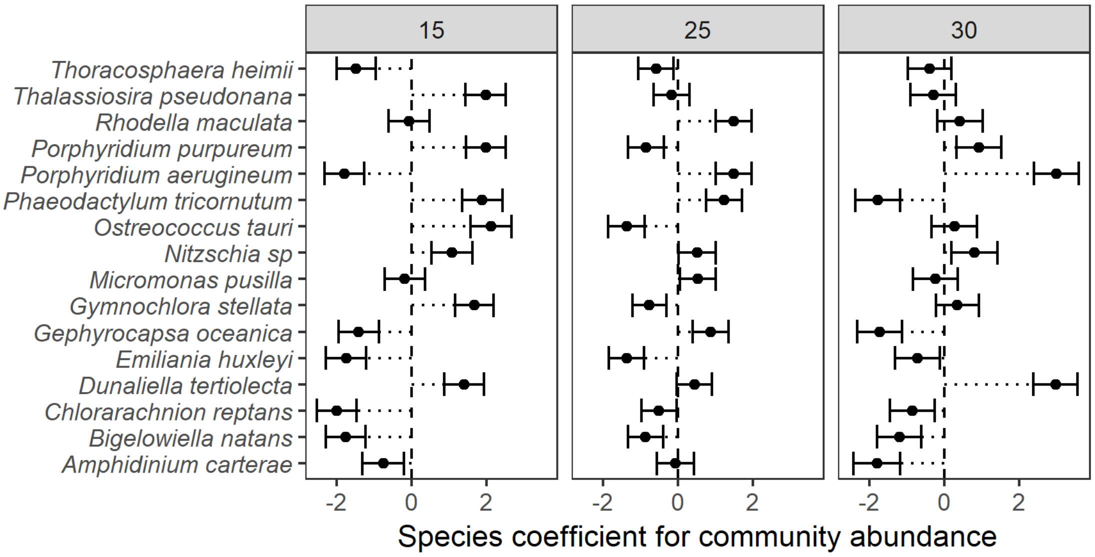

Fig. S3: Species coefficients for abundance by temperature. Impact of species identity on community functioning (community abundance in cells.ml^{-1}) after taking into account the impact of species richness. To take into account the impact of species richness, the model uses the residuals of ln(abundance)~$\log_2$(R) for each temperature as a dependent variable. For each temperature, this dependent variable is modelled against each of the 16 species modelled as a bivariate presence-absence variable. The models explain 50 % of the variance (adjusted R^2) at 15°C, 35 % of the variance at 25°C, and 40% of the variance at 30°C. The figure shows the linear model coefficients for each species at each temperature, with the 95% confidence intervals around each coefficient. Different species have different relative contributions to ecosystem functioning, and these contributions vary with temperature. For instance, *Phaeodactylum tricornutum* has a positive relative contribution to ecosystem functioning at low and medium temperatures, meaning that the addition of this species has a stronger positive effect than the addition of an average species, while the relative contribution turns negative at high temperature, meaning that the addition of the species has a smaller positive impact than average. Conversely, the contribution of *Porphyridium aerugineum* turns from negative to positive when temperature increases. Other species' relative contribution does not change with temperature, as for instance in *Bigelowiella natans* which always has a negative relative contribution. Note that species coefficients vary slightly depending on the metric of ecosystem functioning used, either primary production (Fig. S2), or community abundance (this figure).

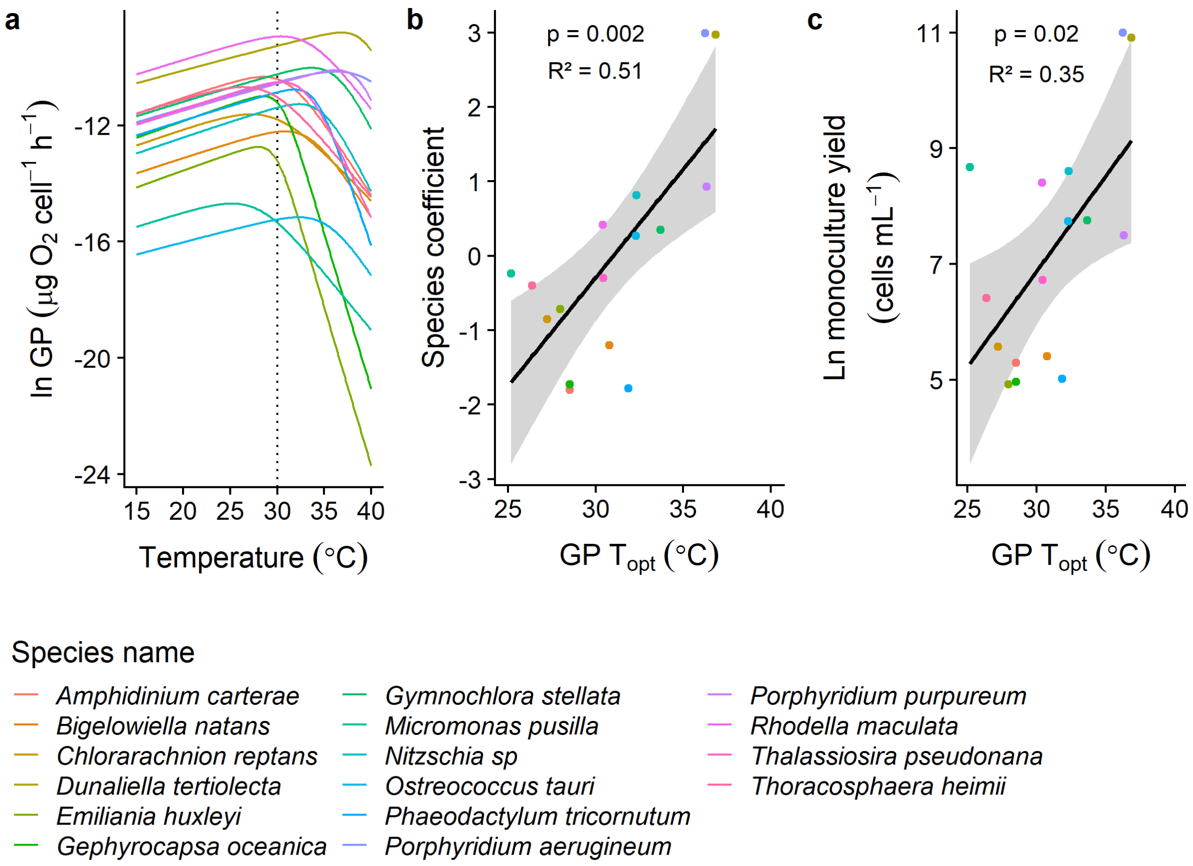

Fig. S4: Link between thermal performance and species contribution to community functioning from the community abundance proxy. (a) Thermal performance curves for gross photosynthesis for each species (see Table S14 for parameters and Fig. S8 for detailed fits for each species). (b) Correlation between species coefficient for community abundance at 30°C and thermal optimum for gross photosynthesis. Species coefficients represent the contribution of each individual species to the community functioning and are calculated from the residuals of the random partitions analysis of the diversity-functioning relationships for abundance (Fig. S3). (c) Correlation between mean species yield in monoculture at 30°C (ln abundance in cells mL^{-1}) and thermal optimum for gross photosynthesis (°C). Analyses reveal that the thermal optimum for gross photosynthesis was strongly correlated with species relative contribution to ecosystem functioning at 30°C (Table S6, Fig. S5b) as well as to the yield of each species in monoculture at 30°C (Table S8, Fig. S6b). The figure is analogous to Fig. 3 from the main document, and shows that the results are congruent regardless of the ecosystem production proxy used.

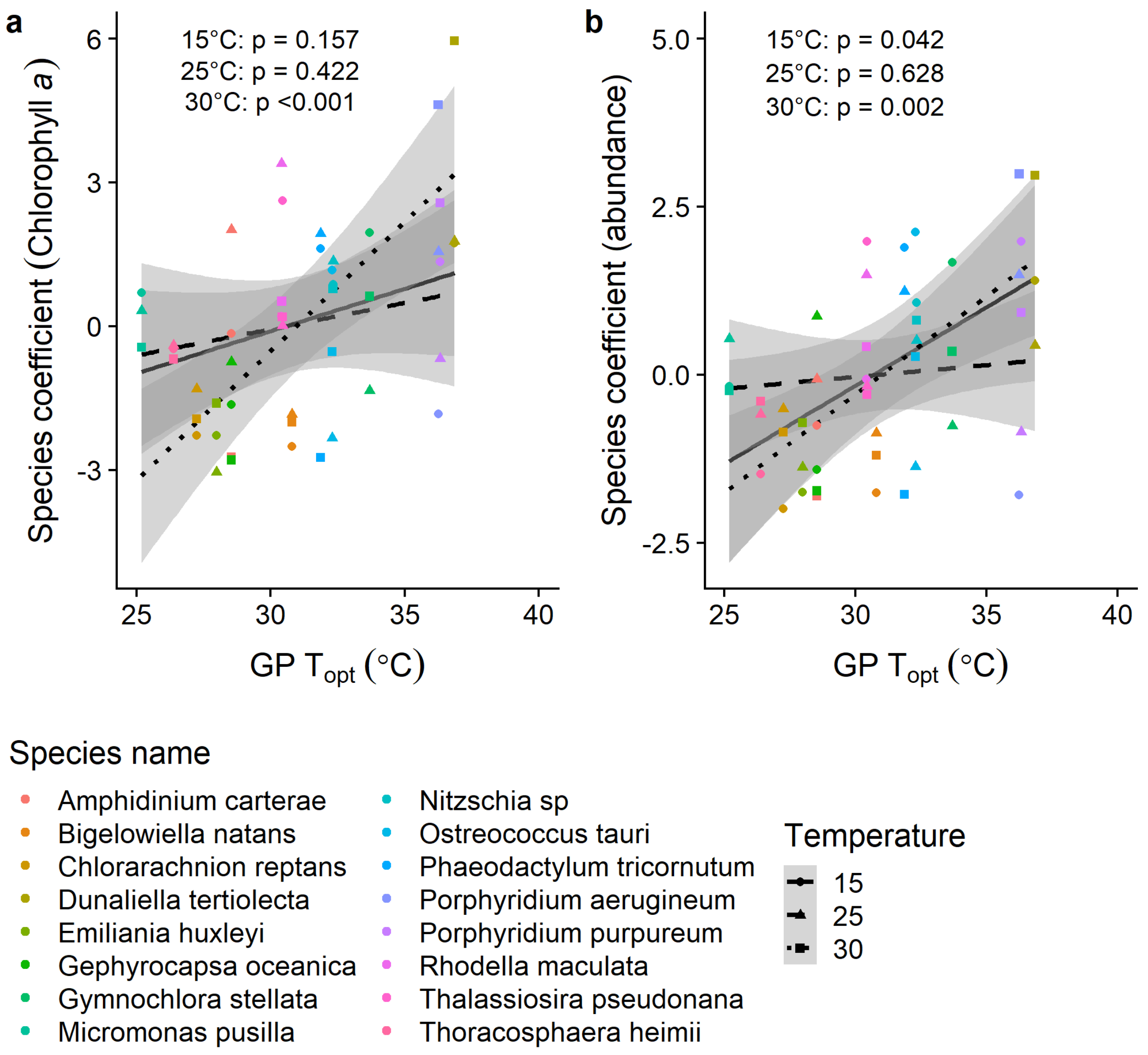

Fig. S5: Link between thermal performance and species relative contribution to community functioning at each of the three temperature levels for (a) the chlorophyll *a* proxy and (b) the abundance proxy. Correlation between species coefficient at 15 (circles, full lines), 25 (triangles, dashed lines) and 30°C (squares, dotted line) and thermal optimum for gross photosynthesis. Positive species coefficients represent species that have a higher than average contribution to ecosystem production, negative coefficients represent lower than average contributions. Thermal optimum for gross photosynthesis has a strong impact on species coefficient at 30°C, but no significant impact at 15 and 25°C on the chlorophyll *a* proxy (see Table S5). This lack of impact of T_{opt} at lower temperatures was to be expected, as the minimum optimum temperature observed for the species is 25.5°C, higher than the two lower temperature treatments. Similar results are found on the abundance proxy, with significant effects at 15°C and 30°C (Table S6).

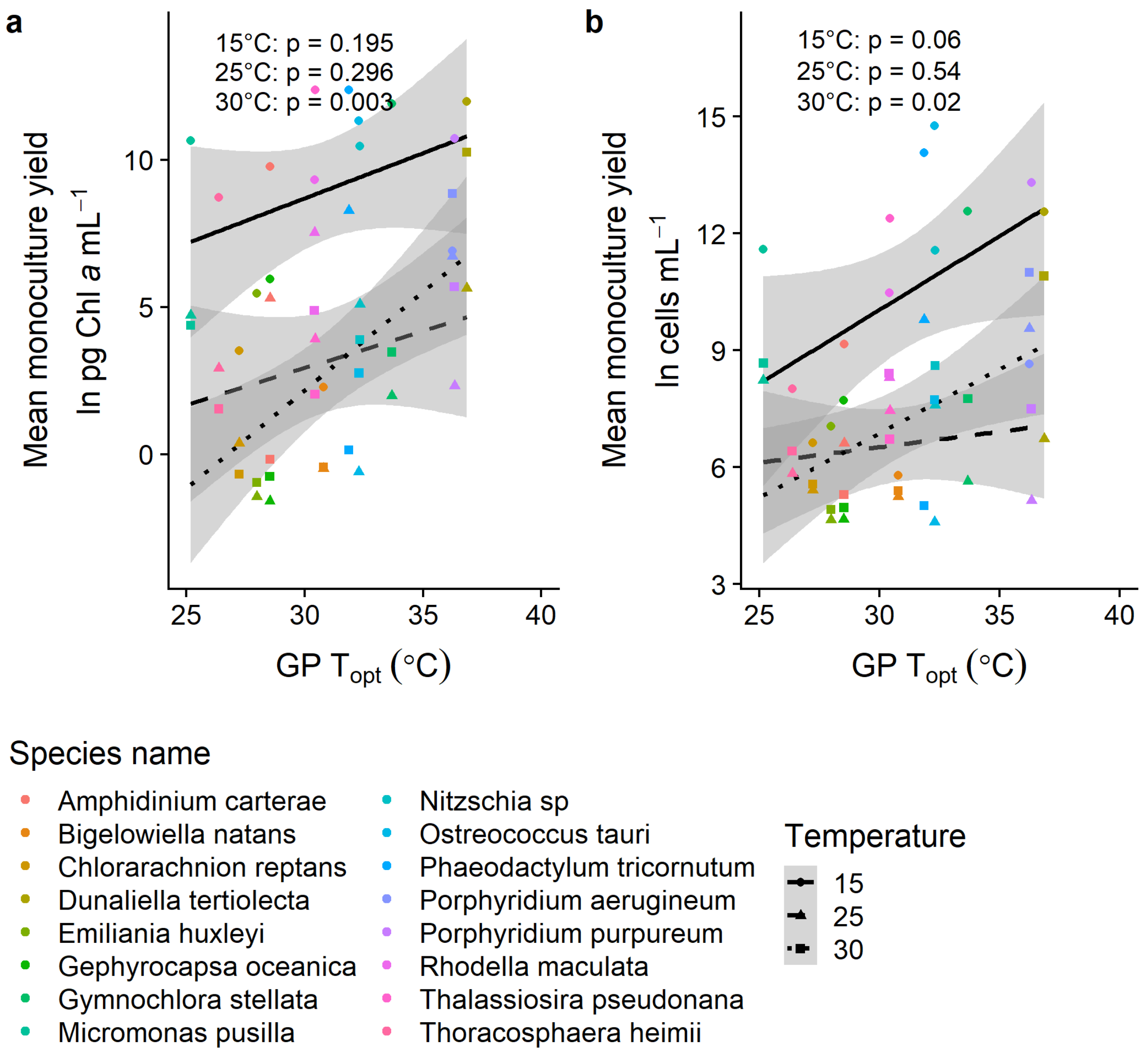

Fig. S6: Link between thermal performance and species yield in monoculture at each of the three temperature levels for (a) the chlorophyll *a* proxy and (b) the abundance proxy. Correlation between yield in monoculture at 15 (circles, full lines), 25 (triangles, dashed lines) and 30°C (squares, dotted line) and thermal optimum for gross photosynthesis. Thermal optimum for gross photosynthesis has a strong impact on species coefficient at 30°C, but no significant impact at 15 and 25°C on both the chlorophyll *a* and the abundance proxies (Table S7, S8). This lack of impact of T_{opt} at lower temperatures was to be expected, as the minimum optimum temperature observed for the species is 25.5°C, higher than the two lower temperature treatments.

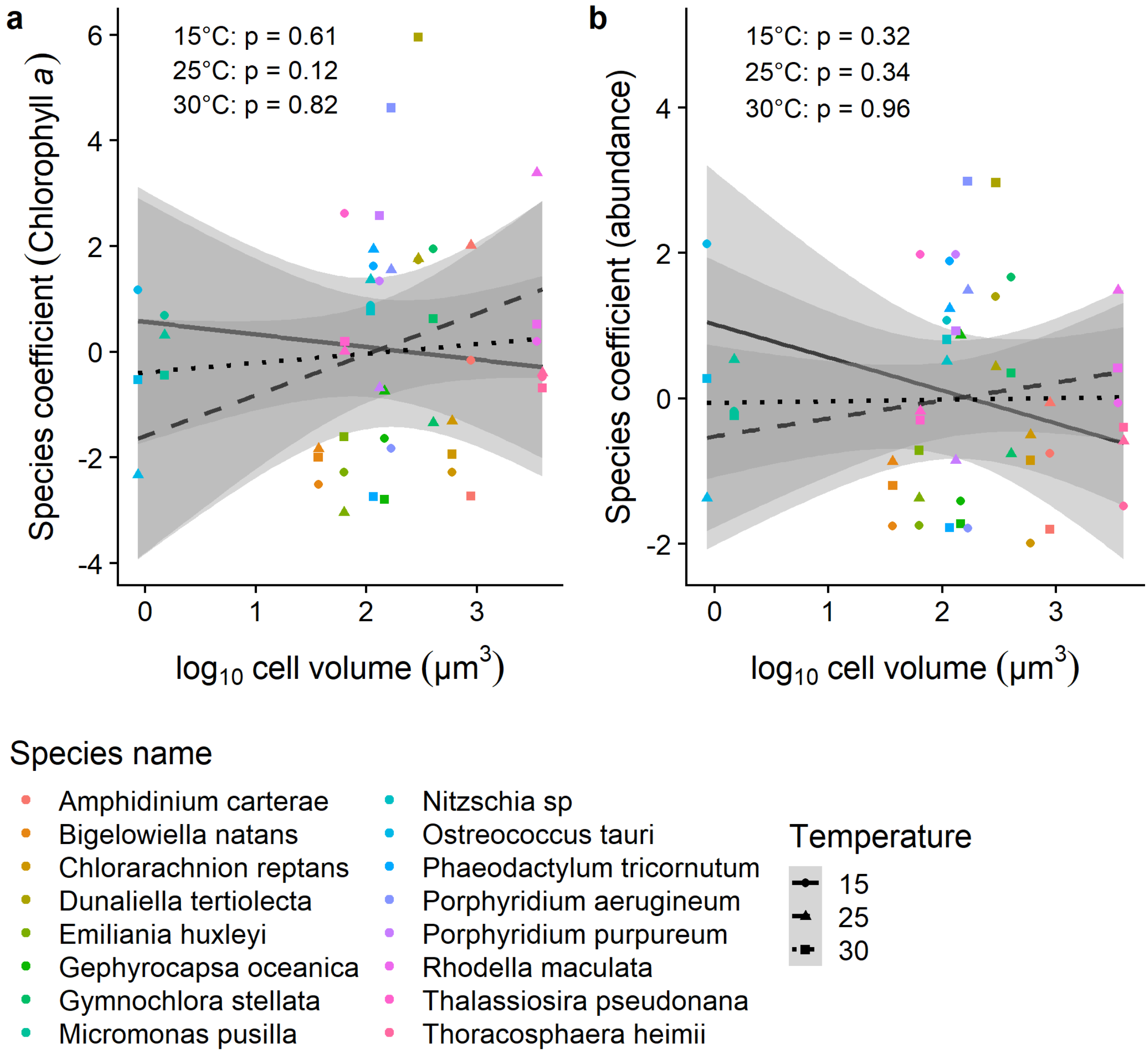

Fig. S7: Link between cell volume and species relative contribution to community functioning at each of the three temperature levels for (a) the chlorophyll *a* proxy and (b) the abundance proxy. Correlation between species coefficient at 15 (circles, full lines), 25 (triangles, dashed lines) and 30°C (squares, dotted line) and thermal optimum for gross photosynthesis. Positive species coefficients represent species that have a higher than average contribution to ecosystem production, negative coefficients represent lower than average contributions. There is no significant relationship between $\log_{10}$(cell volume) and species coefficients at any temperature for any ecosystem function proxy (Table S9, S10).

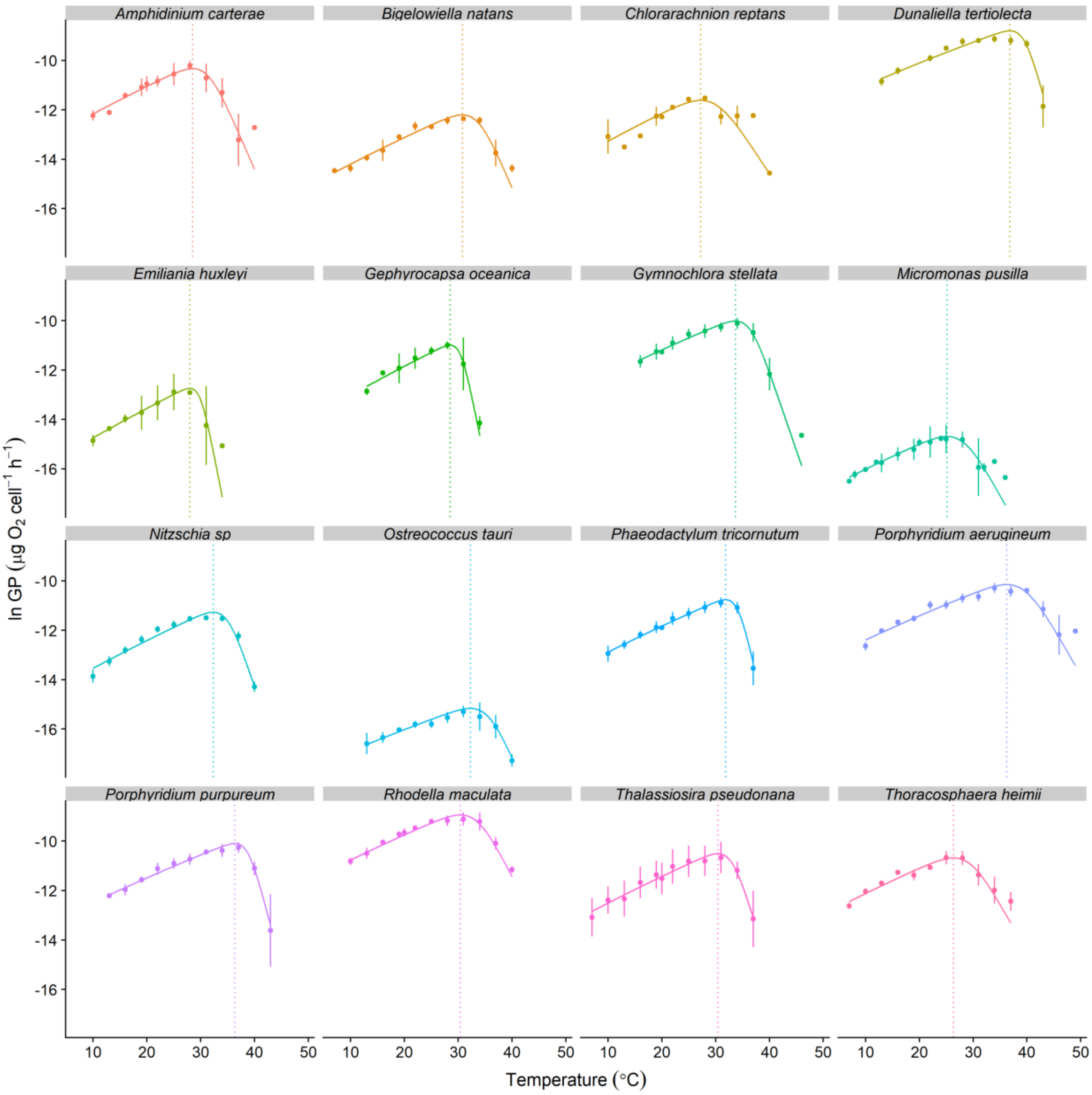

Fig. S8: Thermal performance curves for gross photosynthesis for each of the 16 phytoplankton species. The data points presented are the natural logarithm of mean metabolic, with error bars denoting ± SEM (n = minimum of 3 biological replicates per response for each species). The fitted lines for each species are from the random effects of a non-linear mixed effects model fitted to the rate data using the Sharpe-Schoolfield equation (see Methods and Table S14 for model parameters). The vertical dashed lines correspond with the optimal temperatures for each species.

Supplementary tables

Table S1: Detailed information about the species

Phyla	Species/Strain	Medium	Source location	Cell volume (μm^3)
Chlorophyceae/ Prasinophyceae	*Dunaliella tertiolecta* (CCAP 19/5)	F/2	North Atlantic - English Channel	295.11
	Micromonas pusilla (CCMP1545, RCC 834)	K	North Atlantic - English Channel	1.49
	Ostreococcus tauri (OTH95, RCC 4221)	K	Mediterranean - Gulf of Lion	0.86
Chlorarachniophyceae	*Gymnochlora stellata* (CCMP2057, RCC 626)	F/2	West Pacific Ocean	400.3
	Bigelowiella natans (CCMP621, RCC 623)	F/2	North Atlantic - Sargasso Sea	36.7
	Chlorarachnion reptans (CCAP 815/1 , CCMP239)	F/2	North Pacific - Gulf of California	594.5
Rhodophyceae	*Rhodella maculata* (CCAP 1388/2, SAG 45.85)	F/2	North Atlantic - English Channel	3475.09
	Porphyridium purpureum (CCAP 1380/11)	F/2	Japan	131.62
	Porphyridium aerugineum (RCC 652/ SAG 110.79)	K	North Atlantic - North Sea	166.83
Bacillariophyceae	*Thalassiosira pseudonana* (CCMP 1335)	F/2+Si	North Atlantic - Moriches Bay	63.75
	Nitzschia sp (RCC 80/ ROS97004)	K+Si	North Atlantic - English Channel	109.38
	Phaeodactylum tricornutum (CCAP 1052/1B, CCMP 2558)	F/2+Si	North Atlantic	115.56
Prymnesiophyceae	*Gephyrocapsa oceanica* (RCC 1303/ AC300/ LK7)	K/2	North Atlantic - Arcachon Bay	144.98
	Emiliania huxleyi (CCMP 1516/ CCMP 2090)	K/2	South Pacific Ocean	52.59
Dinophyceae	*Amphidinium carterae* (CCMP 1314)	F/2	North Atlantic - Nantucket Sound	877.18
	Thoracosphaera heimi (AC214/ Nap17 /RCC 1512)	K/2	Mediterranean - Tyrrhenian Sea	3896.8

Table S2: Contrast analysis of the slopes of the biodiversity-ecosystem functioning relationship from model 3 in Table 1 (lm(ln(chlorophyll *a*) ~ temperature * $\log_2$(Richness))). The contrast analysis investigates the slope of the species richness-ecosystem functioning (chlorophyll *a* content) relationship by temperature with the lstrends function from the lsmeans package in R with tukey method of p-value adjustment. See Fig. 2 for a representation of the biodiversity-ecosystem function relationship with the slopes for each temperature.

Contrast	estimate	SE	df	t-value	p-value
15-25	-0.72	0.23	1389	-3.20	0.004**
15-30	-1.41	0.23	1389	-6.23	<0.001***
25-30	-0.69	0.23	1389	-3.03	0.007**

Table S3: Linear models estimating the effect of temperature, species richness and species composition on ecosystem production measured as community abundance. The linear models describe the effect of temperature (T, as a factor), species richness on a log basis ($log_2(R)$), and their interactions on community abundance (ln(cell counts)). At each step, terms are added to the linear model and the residual degrees of freedom (Res. d.f.) and sum of squares (Res. SS) are re-calculated. The treatment degrees of freedom (Treat. d.f), sum of squares (Treat. SS) and F-statistic (F) are calculated at each step only for the term that has been added to the model during that step. R^2 and AIC are calculated for each model. Lower AIC values indicate an improved model. The best model is the model including the interaction between species richness and temperature and it explains 40 % of the variance. See Table S4 for a contrast analysis of the slope of the biodiversity-ecosystem functioning relationship by temperature and Fig. S1 for a graphic representation of the results. This table is analogous to Table 1 from the main document, and shows that the results are congruent regardless of the ecosystem production proxy used.

Step	Model	Res. d.f.	Res. SS	Treat. d.f.	Treat. SS	F	R^2	AIC
0	Intercept	1394	13789.9					7158.8
1	step0+T	1392	10126.6	2	3663.3	251.8	0.27	6732.1
2	step1+$log_2(R)$	1391	8471.7	1	1655	271.7	0.39	6485.2
3	step2+T*$log_2(R)$	1389	8341.8	2	129.9	10.8	0.40	6467.6

Table S4: Contrast analysis of the slopes of the biodiversity-ecosystem function relationship from model 3 in Table S3 (lm(ln(community abundance) ~ temperature * log_2(Richness))). The contrast analysis investigates the slope of the species richness-ecosystem functioning (community abundance) relationship by temperature with the lstrends function from the lsmeans package in R with tukey method of p-value adjustment. See Fig. S1 for a representation of the biodiversity-ecosystem function relationship with the slopes for each temperature. This table is analogous to Table S2, and shows that the results are mainly the same regardless of the ecosystem production proxy used, although we show no significant difference between the slopes at 15 and 25°C on community abundance while the difference is significant on the chlorophyll *a* proxy.

Contrast	estimate	SE	df	t-value	p-value
15-25	-0.18	0.15	1389	-1.20	0.453
15-30	-0.67	0.15	1389	-4.49	<0.001***
25-30	-0.49	0.15	1389	-3.29	0.003**

Table S5: Impact of Topt for gross photosynthesis on species coefficient for chlorophyll a by temperature; summary from linear models in the form of lm(species coefficient for chlorophyll *a* ~ T_{opt}) for each temperature treatment. We provide a representation of the results for the 30°C temperature treatment in Fig. 3b and for all temperature treatments in Fig. S5a.

Temperature	estimate	SE	df	t-value	p-value	R^2
15	0.18	0.12	14	1.49	0.157	0.14
25	0.11	0.13	14	0.83	0.422	0.05
30	0.54	0.13	14	4.29	<0.001***	0.57

Table S6: Impact of Topt for gross photosynthesis on species coefficient for community abundance by temperature; summary from linear models in the form of lm(species coefficient for abundance ~ T_{opt}) for each temperature treatment. We provide a representation of the results for the 30°C temperature treatment in Fig. S4b and for all temperature treatments in Fig. S5b. This table is analogous to Table S5, and shows similar results to the chlorophyll *a* proxy, although there is a significant effect of T_{opt} at 15°C that is not found in Table S5.

Temperature	estimate	SE	df	t-value	p-value	R^2
15	0.23	0.10	14	2.24	0.042*	0.26
25	0.04	0.07	14	0.50	0.628	0.02
30	0.29	0.08	14	3.84	0.002**	0.51

Table S7: Impact of Topt for gross photosynthesis on species yield in monoculture (as chlorophyll a) by temperature; summary from linear models in the form of lm(species chlorophyll *a* in monoculture ~ T_{opt}) for each temperature treatment. We provide a representation of the results for the 30°C temperature treatment in Fig. 3c and for all temperature treatments in Fig. S6a.

Temperature	estimate	SE	df	t-value	p-value	R^2
15	0.31	0.23	14	1.36	0.195	0.12
25	0.25	0.23	14	1.09	0.296	0.08
30	0.67	0.18	14	3.65	0.003**	0.49

Table S8: Impact of Topt for gross photosynthesis on species yield in monoculture (as abundance) by temperature; summary from linear models in the form of lm(species abundance in monoculture ~ T_{opt}) for each temperature treatment. We provide a representation of the results for the 30°C temperature treatment in Fig. S4c and for all temperature treatments in Fig. S6b. This table is analogous to Table S7 and shows that the results are congruent regardless of the ecosystem production proxy used.

Temperature	estimate	SE	df	t-value	p-value	R^2
15	0.38	0.19	14	2.03	0.06	0.23
25	0.08	0.13	14	0.63	0.54	0.03
30	0.33	0.12	14	2.75	0.02*	0.35

Table S9: Impact of cell volume on species coefficient for chlorophyll *a* by temperature; summary from linear models in the form of lm(species coefficient for chlorophyll *a* ~ log_{10}(cell volume)) for each temperature treatment. We provide a representation of the results for all temperature treatments in Fig. S7a.

Temperature	estimate	SE	df	t-value	p-value	R^2
15	-0.24	0.46	14	-0.52	0.61	0.02
25	0.77	0.44	14	1.74	0.10	0.18
30	0.18	0.69	14	0.26	0.80	0.00

Table S10: Impact of cell volume on species coefficient for abundance by temperature; summary from linear models in the form of lm(species coefficient for abundance ~ log_{10}(cell volume)) for each temperature treatment. We provide a representation of the results for all temperature treatments in Fig. S7b. This table is analogous to Table S9 and shows that the results are congruent regardless of the ecosystem production proxy used.

Temperature	estimate	SE	df	t-value	p-value	R^2
15	-0.45	0.42	14	-1.08	0.30	0.08
25	0.25	0.25	14	0.99	0.34	0.07
30	0.02	0.39	14	0.05	0.96	0.00

Table S11: Relationship between focal species abundance in polyculture and its abundance in monoculture by temperature; summary from linear models in the form of lm(ln abundance in polyculture ~ ln abundance in monoculture)) for each temperature treatment. We provide a representation of the results for all temperature treatments in Fig. 4.

Temperature	estimate	SE	df	t-value	p-value	R^2
15	0.64	0.065	76	9.95	<0.001***	0.57
25	0.32	0.062	76	5.18	<0.001***	0.26
30	0.48	0.068	76	7.01	<0.001***	0.39

Table S12: Net and transgressive overyielding for chlorophyll a. Comparison between ecosystem functioning (ln(cholorophyll *a*) in pg.ml^{-1}) of the 16-species monocultures to the ecosystem functioning of all of the monocultures and to the ecosystem functioning of the best functioning monocultures by temperature. Mean and 95% CI (calculated as 1.96*SE) of the 16-species polyculture (N = 15 replicates), of the 16 species in monoculture (N = 16 species * 15 replicates = 240), and of the best functioning monoculture (N = 15 replicates). The yield of the 16-species polyculture is always higher than the mean yield of the monoculture, showing overyielding where biodiversity increases ecosystem functioning due to selection and/or complementarity effect. However, there is no sign of transgressive overyielding where the polycultures would have a greater chlorophyll *a* content than the highest monoculture, suggesting that the impact of richness on ecosystem functioning is due to a selection effect instead of a complementarity effect.

	Yield of the 16-sp polyculture			Mean yield of the monocultures			Yield of the best monoculture			
Temperature	mean	95%CI		mean	95%CI		mean	95%CI		Sp. identity
15	11.59	11.21	11.98	8.98	8.51	9.45	12.36	11.99	12.74	*Thalassiosira pseudonana*
25	8.76	8.23	9.28	3.17	2.65	3.68	8.27	7.63	8.91	*Phaeodactylum tricornutum*
30	12.08	10.92	13.25	2.8	2.23	3.38	10.25	8.97	11.54	*Dunaliella tertiolecta*

Table S13: Net and transgressive overyielding for abundance. Comparison between ecosystem functioning (ln(community abundance) in cells.ml^{-1}) of the 16-species monocultures to the ecosystem functioning of all of the monocultures and to the ecosystem functioning of the best functioning monocultures by temperature. Mean and 95% CI (calculated as 1.96*SE) of the 16-species polyculture (N = 15 replicates), of the 16 species in monoculture (N = 16 species * 15 replicates = 240), and of the best functioning monoculture (N = 15 replicates). The yield of the 16-species polyculture is always higher than the mean yield of the monoculture, showing overyielding where biodiversity increases ecosystem functioning due to selection and/or complementarity effect. Further, at 30°C there is a sign of transgressive overyielding where the polycultures have a greater abundance than the most abundant monoculture. This suggests some form of complementarity effect, which was not present when we looked at chlorophyll *a* content (Table S12). There is no sign of transgressive overyielding at 15 and 25°C. Note that the identity of the best monoculture changes with temperature and with the metric of ecosystem functioning used (see Table S12).

	Yield of the 16-sp polyculture			Mean yield of the monocultures			Yield of the best monoculture			
Temperature	mean	95%CI		mean	95%CI		mean	95%CI		Sp. identity
15	12.51	12.13	12.88	10.4	10.01	10.79	14.77	14.19	15.35	*Ostreococcus tauri*
25	9.98	9.47	10.49	6.59	6.34	6.85	9.79	9.13	10.45	*Phaeodactylum tricornutum*
30	13.1	12.7	13.5	7.18	6.85	7.51	11	10.3	11.7	*Porphyridium aerugineum*

Table S14: Parameters of the thermal performance curves. Species level, and fixed effect estimates of the thermal performance curve for gross photosynthesis, predicted from nonlinear mixed effect modelling. Lower and upper values are from the 95% CI intervals surrounding the fixed effect values. Estimated parameters from the Sharpe-Schoolfield equation (eq. 4) are E_a the activation energy (eV), E_h the deactivation energy (eV) which characterizes temperature-induced decrease in rates above T_h (°K) where half the enzymes have become non-functional and $b(T_c)$ the metabolic rate (GP in μgO_2 $cell^{-1}$ $hour^{-1}$) normalized to an arbitrary reference temperature, here T_c = 20°C (+ 273.15). T_{opt} is estimated by differentiating the Sharpe-Schoolfield equation and solving for global maxima, and is presented here in °C instead of °K to facilitate comparison with the temperature treatments. Thermal performance curves for each species are represented in Fig. 3a and S4a, and in more details in Fig. S8.

	$\ln(b(T_c))$	E_a	E_h	T_h	T_{opt}
Amphidinium carterae	-11.05	0.8	4.98	304.31	28.53
Bigelowiella natans	-13.12	0.77	5.06	306.67	30.79
Chlorarachnion reptans	-12.14	0.81	3.64	303.09	27.24
Dunaliella tertiolecta	-10.11	0.65	7.54	312.63	36.86
Emiliania huxleyi	-13.55	0.86	9.93	303	27.98
Gephyrocapsa oceanica	-11.85	0.84	9.73	303.59	28.52
Gymnochlora stellata	-11.18	0.73	6.21	309.49	33.69
Micromonas pusilla	-14.99	0.73	4.01	301.22	25.18
Nitzschia sp	-12.41	0.8	6.2	307.96	32.33
Ostreococcus tauri	-16.02	0.62	4.89	308.64	32.29
Porphyridium aerugineum	-11.42	0.7	4.09	312.62	36.25
Porphyridium purpureum	-11.49	0.71	7.97	311.9	36.33
Phaeodactylum tricornutum	-11.83	0.75	8.66	307.21	31.87
Rhodella maculata	-9.75	0.73	4.37	306.51	30.41
Thoracosphaera heimii	-11.1	0.73	3.98	302.44	26.37
Thalassiosira pseudonana	-11.43	0.77	6.59	306.04	30.43
Fixed effect	-12.09	0.75	6.12	306.7	30.97
95% CI of fixed effect	[-12.88,-11.29]	[0.69,0.81]	[4.9,7.33]	[304.88,308.53]	

Table S15: Spread of optimum values compared to natural spreads in optimum values locally. Comparison between the spread in thermal optimum values of the species chosen in this experiment and the spread in thermal optimum values of marine phytoplankton species found within 1 (resp. 0.1) degree of latitude in a recent meta-analysis (Thomas *et al.* 2012). We extracted the data from supplementary tables S5 and S6 from Thomas et al, giving values for thermal optimum of multiple phytoplankton taxa found across the globe and their precise latitudinal provenance. We rounded the latitudinal values to 1 degree of latitude (resp. 0.1 degree of latitude), and calculated the spread in thermal optimum values as the difference between the minimum value of T_{opt} and the maximum value of T_{opt} from species within the same degree (resp. 0.1 degree) of latitude, as well as the standard deviation in the values of T_{opt} from species within the same degree (resp. 0.1 degree) of latitude. Because multiple latitudinal values had only 1 or 2 observations, we dropped these observations to get only latitudinal values (either 1 degree or 0.1 degree of latitude) for which we had at least 3 observations as there is no possible spread to be calculated on one value, and a spread between two values would be dubious. We then averaged the spread in thermal optimum and the SD in thermal optimum across the latitudinal values to compare it with the spread in thermal optimum values of the species chosen in this experiment. We also redid the analysis with more stringent cut-offs in the minimum number of observations to calculate a spread or a SD, to have at least 4 (resp. 5) observations. We can see that the spread between the minimum and maximum values of thermal optimum within our experiment (11.68°C) is similar to the spread in values of thermal optimum within one degree of latitude (between 8.73 ± 4.81 and 11.43 ± 4.84 °C of spread depending of the stringency of the cut-off value) and even similar to the spread in thermal optimum within 0.1 degree of latitude 7.51 ± 4.75 and 11.8 ± 5.14 °C). The results are similar when we compare values of standard deviation of thermal optimum (see below). This suggests that despite the wide geographic breadth in the provenance of our species, it would be possible to find such a wide range of thermal optimum values

Type of spread	minimum number of observations per latitudinal value	Spread in T_{opt}		SD in T_{opt}		n latitudinal observations
		mean	sd	mean	sd	
Spread within Thomas et al. data						
Spread within 1 degree	3	8.73	4.81	3.3	1.24	17
Spread within a 10th of degree	3	7.51	4.75	3.2	1.59	15
Spread within 1 degree	4	9.96	5.06	3.41	1.27	12
Spread within a 10th of degree	4	10.73	5.3	3.63	1.59	6
Spread within 1 degree	5	11.43	4.84	3.71	1.2	9
Spread within a 10th of degree	5	11.8	5.14	3.79	1.72	5
Spread within our experimental data	-	11.68	-	3.59	-	16
